# Large-scale investigation of dry orthogonal cutting experiments Ti6Al4V and Ck45

**DOI:** 10.1007/s00170-024-14597-2

**Published:** 2024-10-24

**Authors:** Hagen Klippel, Stefan Süssmaier, Nanyuan Zhang, Michal Kuffa, Konrad Wegener

**Affiliations:** Institute of Machine Tools and Manufacturing (IWF), Department of Mechanical and Process Engineering, ETH Zürich, Leonhardstrasse 21, 8092 Zürich, Switzerland

**Keywords:** Dry orthogonal cutting, Material characterisation, AISI 1045, Ti6Al4V

## Abstract

The numerical simulation of metal cutting processes requires material data for constitutive equations, which cannot be obtained with standard material testing procedures. Instead, inverse identifications of material parameters within numerical simulation models of the cutting experiment itself are necessary. This report presents the findings from a large-scale study of dry orthogonal cutting experiments on Ti6Al4V (Grade 5) and Ck45 (AISI 1045). It includes material characterization through microstructural analysis and tensile tests. The study details the measurement of cutting insert geometries and cutting edge radii, evaluates process forces, deduces friction coefficients and coefficients for Kienzle’s force model, and analyzes chip forms and thicknesses as well as built-up edge formation depending on the process parameters. The collected data, stored in pCloud, can support other researchers in the field, e.g. for recomputations within numerical models or inverse parameter identifications. The dataset includes force measurements, cutting edge scans, and chip images including longitudinal cross-sections of chips.

**Supplementary Information:**

The online version contains supplementary material available at 10.1007/s00170-024-14597-2.

## Introduction

The metal cutting process is characterized by harsh conditions, which comprise large plastic strains up to 700%, strain rates up to $10^6s^{-1}$, and temperatures ranging from 500 to 1400 $^\circ$C, according to [1]. In the numerical simulation of such processes, material parameters for constitutive models are required, but these conditions cannot be reproduced neither in simple (direct) experiments nor in experiments combining the aforementioned conditions. Instead, material parameters need to be deduced from an inverse identification, where the cutting test itself serves as a material test, as for example shown in [2] for Ck45 material, in [3] for Al6061-T6 material, or in [4] for Ck45 and 16MnCr5 material.

Constitutive model constants taken from literature, e.g. for Ti6Al4V [5], show large variations, and so do the simulation results. Raw materials specified by the respective standards, when sourced from different suppliers and charges, show slight variations in the chemical composition within the tolerance bands and may have undergone different manufacturing steps (drawing, rolling, forging) and heat treatments, which finally leads to a wide scatter of thermo-mechanical behaviour [6]. An investigation performed on ferritic-pearlitic steels in [7] shows the yielding behaviour of C45 with a strong dependency of the microstructure. Similar variations are shown in [8], where the influence of the chemical composition and the manufacturing process on the yielding behaviour is analyzed. Since the initial conditions of the materials are often not clearly indicated in the literature, it is difficult to select a suitable constitutive model parameter set from literature for the numerical simulation of documented experiments. For example, [9] documents process forces and chip geometry measurements for Ti6Al4V, but properties for their material batch are not given. Similarly, [10] documents orthogonal cutting test results for various process parameter combinations without properties, which makes it difficult to recompute the experiments within numerical simulations.

**Fig. 1:**
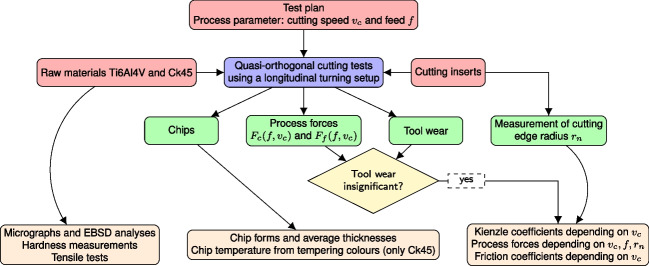
Flow chart of the investigations in this work with inputs (red boxes), outputs (green boxes), and result evaluations (orange boxes)

Ck45 is a perlitic-ferritic steel which is easy to machine and widely used in mechanical engineering applications, e.g. for crankshafts, bolts, gears, and bearings [11]. The material exhibits dynamic strain aging (DSA), also known as blue brittleness, at elevated temperatures leading to increased yield stresses depending on plastic strain rate and temperature and thus can affect the chip formation and process forces. In [12], the importance of considering DSA effects in the constitutive modelling is highlighted, where improvements in the metal cutting of Ck45 are shown. A constitutive model which considers DSA is successfully presented in [13], and in [14], improvements of cutting force and chip formation predictions are shown when using constitutive models considering the DSA.

Titanium alloys are of great industrial interest [15] and find wide use in biotechnical applications due to their biocompatibility [16] and are also popular in aeronautics [17] due to their low density and high strength. The most widely used titanium alloy is Ti6Al4V which however is considered difficult to machine because the material removal rate is low if the tool wear is to be low, and since the high strength causes high process forces, crater wear forms close to the cutting edge due to short chip contact lengths and the low thermal conductivity supports segmented chip formation, which can cause vibrations.

It can be concluded from the above that it is not only necessary to document the process forces, but also to characterize the raw materials and the evolution of process forces, friction coefficients, chip thicknesses, and chip shapes as a function of the process parameters on the same batch of material. Therefore, the aim of the present publication is the documentation of a large-scale testing programme of Ck45 (AISI 1045) and Ti6Al4V (3.7165, Grade 5) using dry quasi-orthogonal cutting experiments together with a characterization of the raw materials. The cutting tests are performed in a wide range of feed rates ($f=0.01..0.4 mm/rev$) and cutting speeds ($v_c=10...500 m/min$). Prior to the cutting experiments, the raw materials are examined: hardness tests, as well as microstructural analyses by means of investigating etched samples and electron backscatter diffraction (EBSD) analyses, are conducted to detect any irregularities, e.g. preferential grain orientations or material anisotropies, that may be caused by the manufacturing process. Tensile tests are carried out on test specimens of these materials, and material parameters for quasi-stationary conditions, together with the rate dependency at low strain rates, are documented.

In the orthogonal cutting tests, neither lubrication nor cooling has been used. Each cutting test was performed with an unused cutting edge to minimize possible wear effects on the process. All cutting edges were measured using 3D metrology before the cutting tests to determine the cutting edge radii in the unworn state using the procedure according to [18]. This is necessary because the cutting edge radius can have a significant influence on the process forces as reported by [19] for steel and in [20] for Ti6Al4V. The measured process forces, chip shapes, average chip thicknesses, chip microstructures, apparent friction coefficients according to Merchant [21] as well as an estimation of the friction coefficients according to Albrecht [19] and, where applicable, built-up edge formations and tempering colors are documented. The measured process forces are used to derive Kienzle coefficients for both materials over a large range of cutting speeds. The results of this investigation shall provide a database which can be used for recomputations within numerical simulations.

**Fig. 2:**
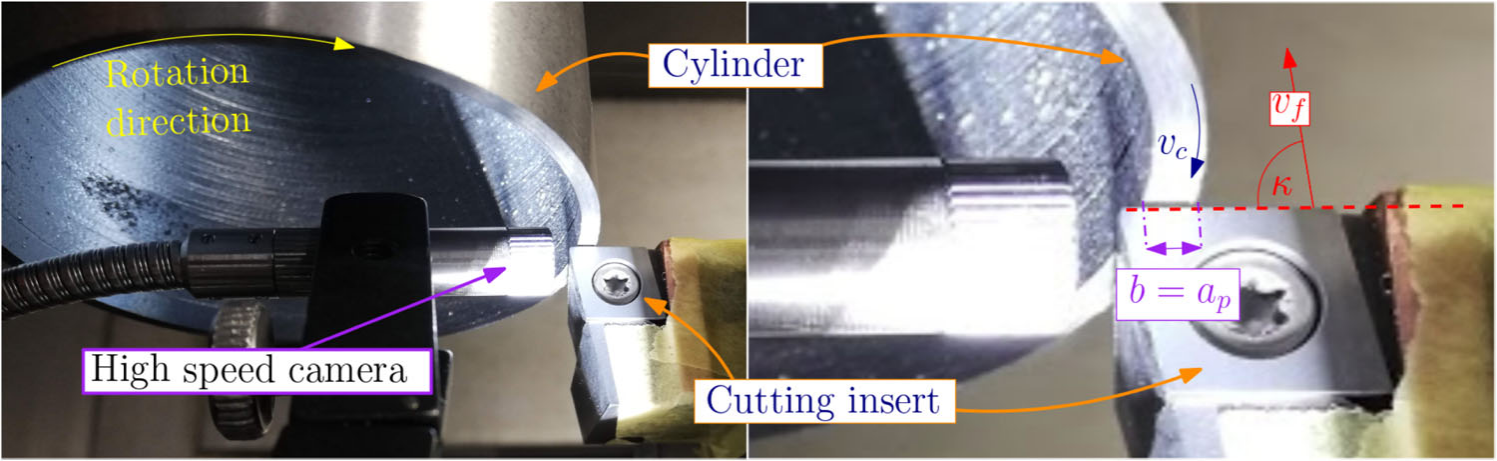
Setup of the quasi-orthogonal cutting experiment: cylinder with cutting insert (left) and close-up (right) with cutting width *b*, feed speed $v_f$, and cutting speed $v_c$ directions

**Fig. 3:**
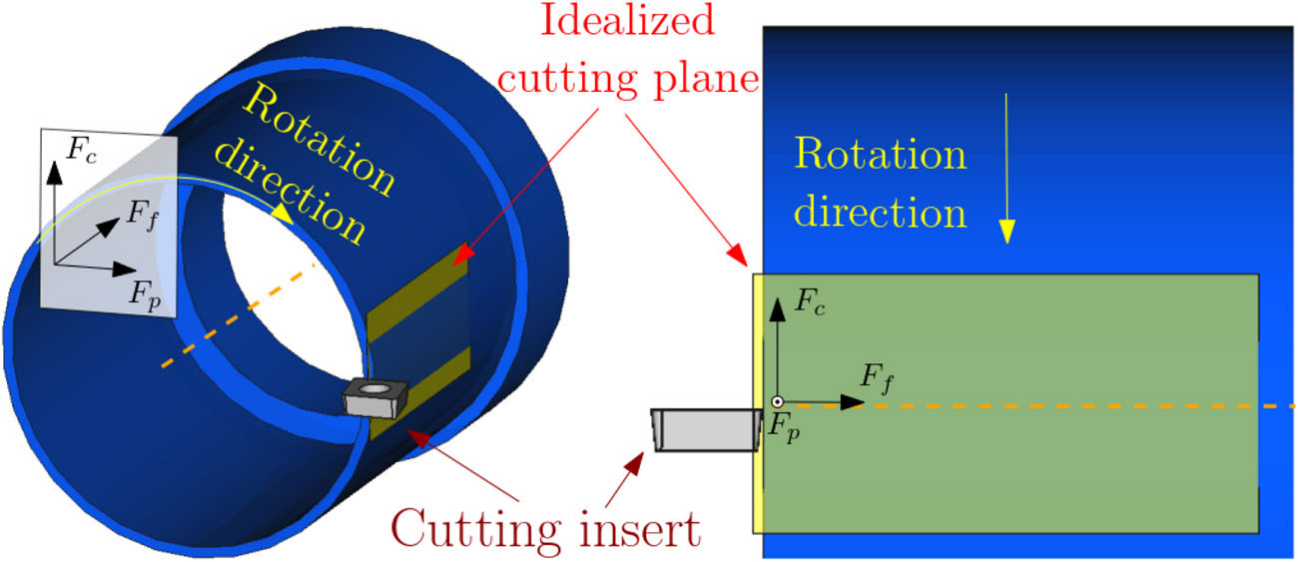
Quasi-orthogonal cutting setup with process force components: cylinder with cutting insert (left) and cutting plane in 2D (right)

## Materials and methods

A flow chart of this investigation is provided in Fig. 1. Basic inputs to the orthogonal tests are the raw materials, cutting inserts, and the test plan. The raw materials are used for micrographs, EBSD analyses, hardness measurements, and tensile tests. The cutting inserts are investigated before the tests to determine the cutting edge radii distribution along the cutting edge length. Output from the cutting tests are the chips, process forces, and the tool wear. The chips from each experiment are used to determine the average chip thicknesses, and from Ck45 chips, the tempering colours are investigated to estimate the chip temperature. The tool wear is analyzed qualitatively. If the tool wear is not high, the force measurements are used to analyze the frictional behaviour, to determine the Kienzle coefficients, and to evaluate the process forces with regard to feed rates, cutting speeds, and cutting edge radii. In the following sections, the methodologies and results are explained in detail.

### Cutting test setup

For the machining test, thin-walled cylinders with a diameter of $D \approx {72}\,{\text {mm}}$ and a wall thickness of $d \approx {2}\,{\text {mm}}$ are turned longitudinally on a Schaublin 42 L CNC lathe. This emulates an orthogonal cut closely. The cylinders are turned inside and outside from the raw workpieces in the same clamping setup. The machining of the outer surfaces removes the scaling and the decarburated zone of the Ck45. The setup with a cylinder in place is shown in Fig. 2 together with the experimental setup for the orthogonal cutting tests.

Figure 3 shows the forces acting on the cutting insert.

Forces are measured using a Kistler 9121A5 dynamometer in combination with a Kistler 5019A charge amplifier and digitalized with a NI USB-6211 data acquisition system. The signal is low-pass filtered with a cut-off frequency of 30 Hz before being sampled with 1.1 kHz. Since the force measurement uses the piezoelectric effect, some voltage drift occurs over time, which leads to small offsets in the measured force signal $\tilde{F}_{meas}$. The force offset $\tilde{F}_{offset}$ overlays the measured process force $\tilde{F}_{proc}$ and is corrected after each cutting experiment:

1 $$\begin{aligned} \tilde{F}_{proc} = \tilde{F}_{meas} - \tilde{F}_{offset} \end{aligned}$$

**Fig. 4:**
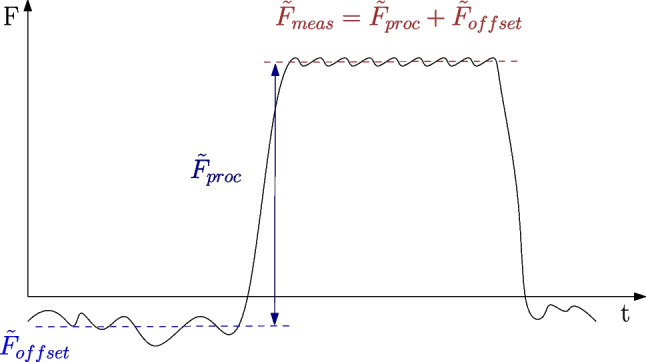
Force measurements using a piezoelectric dynamometer are overlayed with small force offsets due to voltage drifts and are corrected by evaluating the idle forces $\tilde{F}_{offset}$ before the tool contacts the workpieces

The offset $\tilde{F}_{offset}$ of the force is evaluated by positioning the cutter before the cut with a distance of 1 mm away from the cylinder to cut. With the desired feed rate of the experiment, the cutter approaches the workpiece, and during this time, the idle forces $\tilde{F}_{offset}$ are recorded and then used to correct to force signals with Eq. 1. A schematic of this correction is shown in Fig. 4.

High-speed camera recordings are conducted for the first cutting tests with a Phantom V12.1 camera using an endoscope (see the cutting setup in Fig. 3). References to these records are linked in the result tables in the appendix Tables 17 and 18. In order to simplify numerical modelling and to reduce the number of unknown parameters, the experiments are performed as dry cuts. Each combination of feed rates *f* and cutting speed $v_c$ is usually tested three times to ensure results quality and repeatability. For each test, an unused cutting edge is used and test durations are kept short in an attempt to keep tool wear insignificant.

#### Test plan

**Fig. 5:**
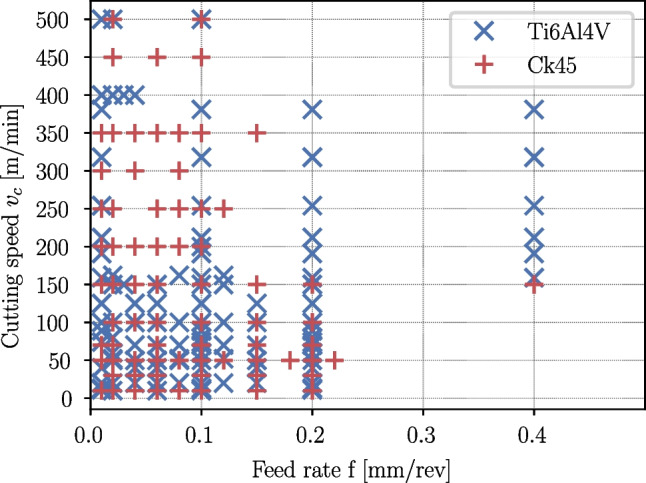
Process parameter combinations used in the orthogonal cutting experiments

Orthogonal cutting tests are conducted for a large range of feed rates and cutting speeds. Each experimental set is repeated three times to ensure the reproducibility of the results. Because it is not clear, before the cutting experiments, when significant tool wear will occur, the cutting speeds $v_c$ and feed rates *f* are gradually increased until the process forces become instable or noticeable tool wear occurs. The process forces of such experiments are not analyzed. In total, 520 cutting tests are conducted, of which 288 are for Ti6Al4V and 232 for Ck45. The parameter ranges are given in Table 1, and all combinations of feed rates *f* and cutting speeds $v_c$ which are used in the cutting experiments of Ti6Al4V and Ck45 are displayed in Fig. 5.

**Table 1:** Test matrix of the cutting tests

	Cutting speed	Feed rate
Material	$v_c (m\!/\!min)$	*f*(*mm*/*rev*)
Ti6Al4V	10...500	0.01...0.4
Ck45	10...500	0.01...0.4

### Cutting inserts

**Fig. 6:**
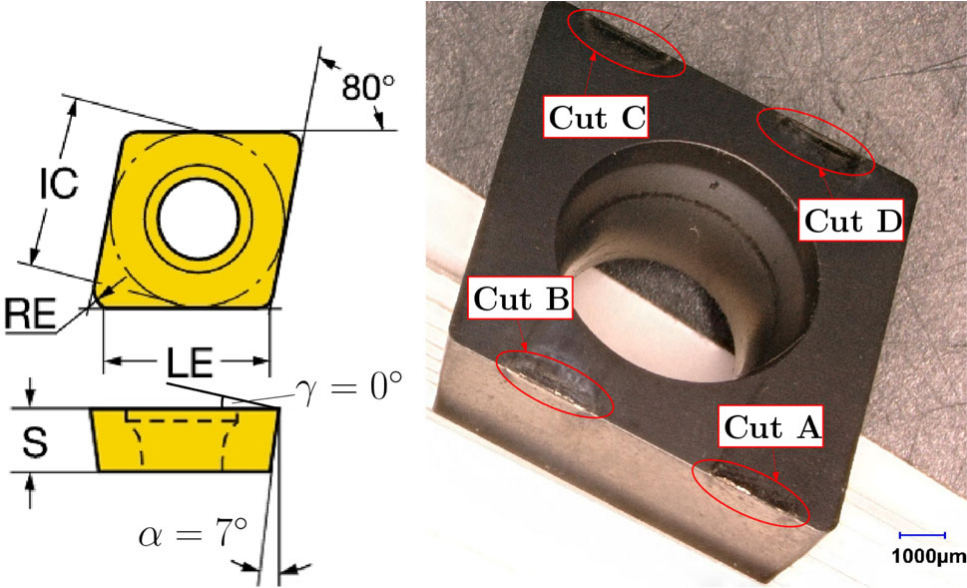
Cutting insert geometry (left) from [23] and four cut positions A–D (right) for cutting experiments, from [24]

Uncoated turning inserts *Sandvik Coromant* CCMW 09T304 H13A (ISO) are used for the cutting experiments. The material of the cutting tool insert is grade H13A, which is a cobalt-bonded tungsten carbide with a Co content of $\approx 6wt\%$ and a WC content of $94wt\%$ [22]. The main geometrical data of the inserts are given in Table 2, and pictures of the turning insert geometry are provided in Fig. 6.

**Fig. 7:**
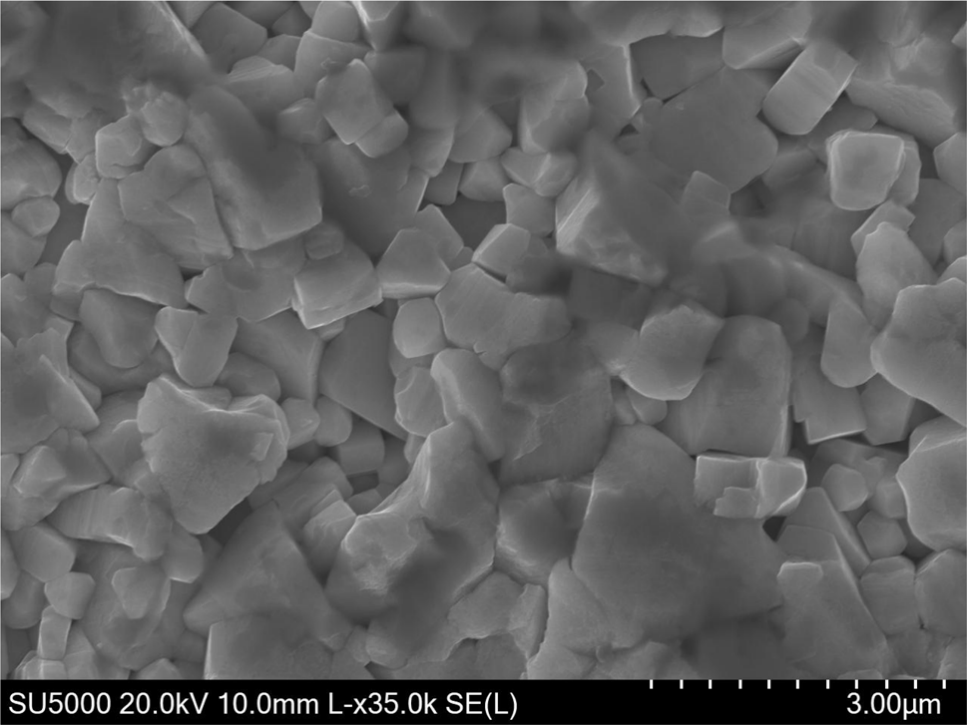
SEM analysis of the rake face prior to experiments on one of the *Sandvik Coromant* CCMW 09T304 H13A (ISO) inserts. Grain sizes are visible in the order of 1 to 3 $\mu m$

One of the inserts was analyzed prior to the cutting experiments using a scanning electron microscope (SEM) and is shown in Fig. 7. The grain sizes are in the order of 1–3 $\mu m$. The hardness is measured on the rake face at three different positions using micro-hardness measurements and hardnesses between 1812 to 1877 HV10 are obtained.

Additionally, surface roughnesses are measured on the rake face and the clearance face on one of the cutting inserts. The measurement is performed according to ISO 4287 using confocal measurements of the cutting insert surface topology with a Sensofar S neox microscope with 150$\times$ magnification. The results are compiled in Table 3.

**Table 2:** Main geometry data of *Sandvik Coromant* CCMW 09T304 H13A (ISO) inserts

Dimension	Symbol	Value
Corner edge radius	RE	${0.397}\,{\text {mm}}$
Cutting edge height	S	${3.969}\,{\text {mm}}$
Inscribed circle	IC	${9.525}\,{\text {mm}}$
Cutting edge length	LE	${9.272}\,{\text {mm}}$
Clearance angle	$\alpha$	$7^{\circ }$
Rake angle	$\gamma$	$0^{\circ }$
Cutting edge radius	$r_n$	See Section 2.2.1

**Table 3:** Measured surface roughnesses of rake and clearance face according to ISO 4287

Face of cutting insert	$R_a (\mu m)$	$R_z (\mu m)$
Rake face	0.295	1.956
Clearance face	0.289	1.850

**Fig. 8:**
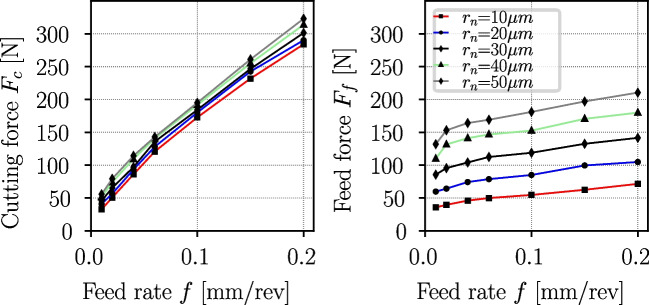
Influence of cutting edge radius $r_n$ and feed rate *f* on the cutting and feed forces for orthogonal cutting of Ti6Al4V, from [20]. The forces are normalized to a cutting width of $b={1}\,{\text {mm}}$, the cut speed was $v_c={70}\,{\text {m/min}}$, the rake angle $\gamma =10^\circ$, and the clearance angle $\alpha =8^\circ$

The cutting edge radii along the cutting edge length LE exhibit a certain scatter which results from the sintering and brushing process. Since these radii have a significant impact on the process forces, they are optically measured (see details in chapter 2.2.1). An Applitec SCACL-2020X-09 tool holder (ISO-2216) is used, which has an entering angle $\kappa$ = 90° and an inclination angle $\lambda$ = 0°. With these settings, the back engagement is $a_p = b \cdot sin \kappa = b$ and the uncut chip thickness is $h = f \cdot sin \kappa = f$. Each insert is used for four cutting experiments (two cuts per side of the insert) where after every experiment another position A–D is used on the insert. These four cut positions A–D are shown in Fig. 6.

#### Determination of cutting edge radii

The shape of the cutting insert, especially the cutting edge radius, has a major influence on the process forces of the turning operation. Albrecht [19] showed for steel that with increasing cutting edge radius, the feed force $F_f$ increases strongly, and the cutting force $F_c$ increases moderately. The effect becomes more pronounced with higher feed rates. Wyen [20] conducted a similar investigation for Ti6Al4V revealing comparable influences on the process forces (see Fig. 8).

**Fig. 9:**
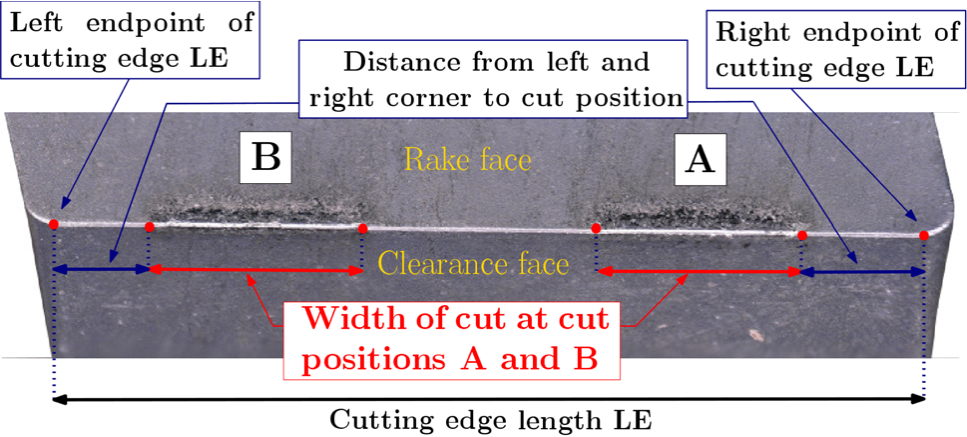
Cutting insert with cut positions A and B showing start and endpoints of the cutting edge, shortest distances from the corners to the beginning of the cutting zones, and width of cuts

The inserts in this investigation have a cutting radius unspecified by the manufacturer, and therefore, all cutting edges (2 $\times$ 149 inserts) of the cutting inserts are optically measured with an Alicona InfiniteFocusG4 microscope prior to the cutting tests in an unused condition. With the focus variation principle [25, 26], 3D data of the scanned surfaces are generated, where a 20$\times$ magnification with 0.5 µm vertical and 7 µm lateral resolution is used to scan along the complete cutting edge length (LE). Initially, a scan depth of 60 µm was used, but since in some positions cutting edge radii of about 50 µm appeared, it was then later increased to 400 µm. Before the cutting edge radii determination the scanned 3D data is reworked. In a first step, single peaks (measurement artefacts) are removed. This is followed by the determination of the left and right endpoints of the cutting edge (LE) between the corner radii for which height profile gradients are evaluated. In between these two points, the cutting edge radii are analyzed and they serve as reference points for the exact determination of the cut position after the experiments (Fig. 9).

**Fig. 10:**
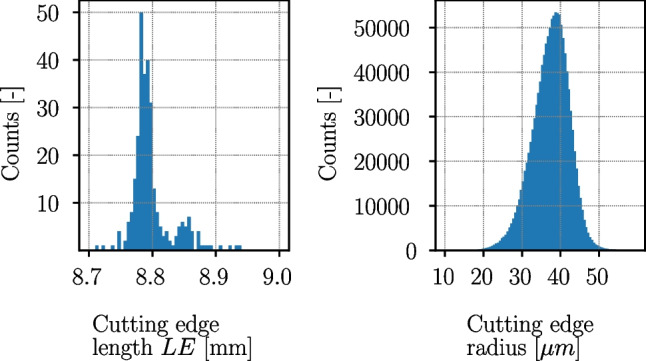
Histogram of cutting edge lengths *LE* (left) and cutting edge radii (right) over all cutting inserts

**Fig. 11:**
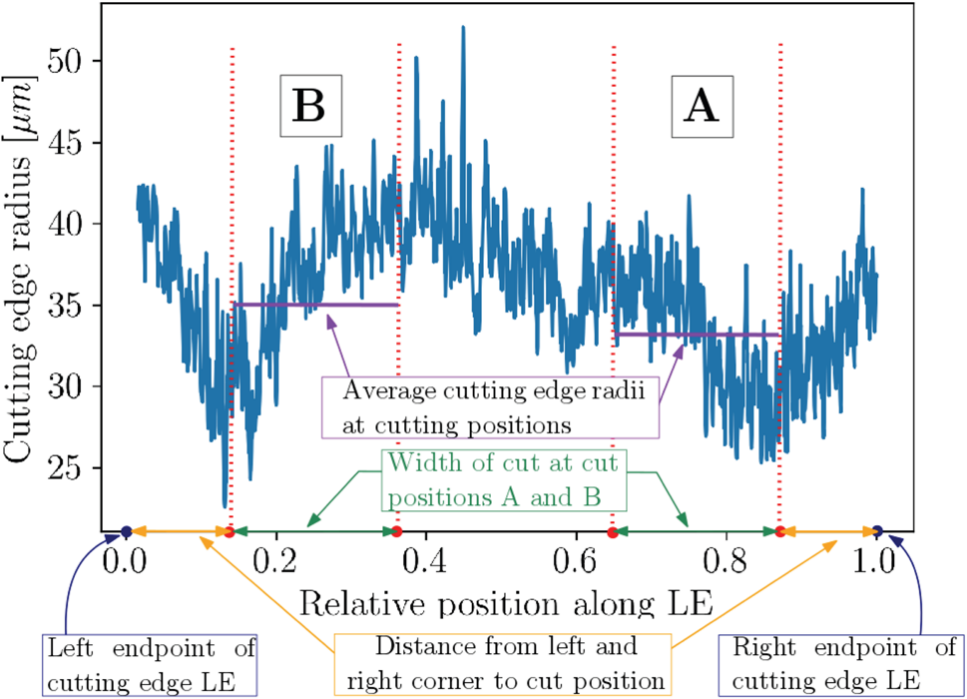
Example of cutting edge radii variation along a single cutting edge, with averaged cutting edge radii at cut positions A and B

The determination of the cutting edge radii follows the procedures outlined in [18, 27] in order to ensure the reproducibility of the results. A total of about 1,275,000 cutting edge radii ($>4000$ radii per cutting edge) are extracted from the scans. Over all 149 cutting inserts, the cutting edge radius along LE varies between $\approx$ 20 µm and $\approx$ 50 µm (Fig. 10). The variation of the cutting edge radius along one cutting edge length *LE* can be significant, as for instance shown in Fig. 11, where it varies between $r_n \approx$ 25 µm and $r_n \approx$ 50 µm. The mean cutting edge radius is 37.5 µm with a standard deviation 4.9 µm. The scatter of the radius is the reason, why the exact cutting edge radius was determined after the experiments, when the location of the cut on the cutting edge is known for every insert and its four cut positions A–D. Histogram plots of the cutting edge length *LE* and the cutting edge radii are given in Fig. 10. The length of the cutting edge *LE* varies between $\approx$ 8.7 mm and $\approx$ 8.95 mm which is inline with the tolerance class of the selected inserts. The cutting edge radius is averaged along the cut width of $\approx$ 2 mm for each experiment and is given in Tables 17 and 18 together with the respective standard deviation.

### Chip thickness measurements

**Fig. 12:**
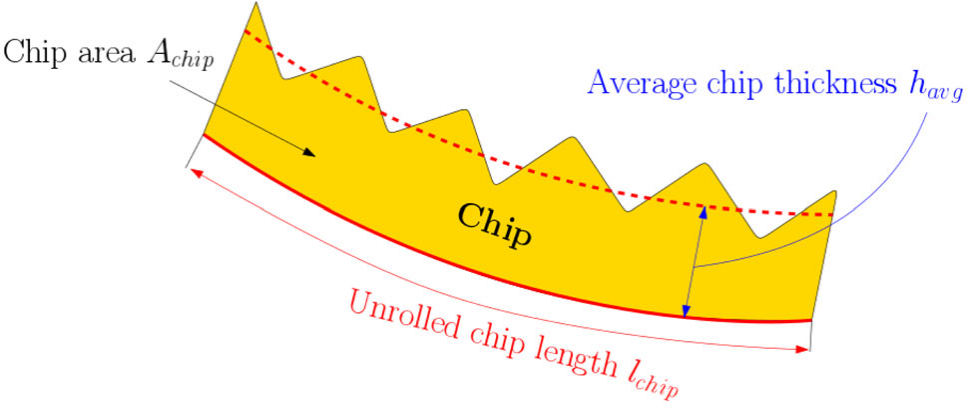
Measurement of average chip thicknesses $h_{avg}$ by the chip area $A_{chip}$ and unrolled chip length $l_{chip}$

At least one chip from the three repetitions of each set of feed rate *f* and cutting speed $v_c$ is embedded in Bakelite, ground (120, 240, 500, 1000, 2500, 4000) and polished ($6\mu m$, $3\mu m$, $1\mu m$). After polishing, the chips are etched with Kroll (Ti6Al4V) and Nital (Ck45). The geometry and microstructure are then analyzed with a Keyence VHX-5000 microscope. The main dimensions which are measured are the chip area $A_{chip}$ and the unrolled chip length $l_{chip}$ using a polygonal approximation (see Fig. 12) from which the average chip thickness $h_{avg}$ is computed as follows:

2 $$\begin{aligned} h_{avg} = \frac{A_{chip}}{l_{chip}} \end{aligned}$$

Due to the embedding process in Bakelite under high pressure, the chip curling radii are not evaluated, as thinner chips in particular are deformed thus changing the curvature.

### Delivery condition of the materials

Raw materials of Ck45 and Ti6Al4V are used in cylindrical form with a diameter of $\approx$ 80 mm and a height of 90 mm. All cylinders are from the same batch of the respective material.

Ti6Al4V consists of two phases: $\alpha$-phase and $\beta$-phase. The $\alpha$-phase is stabilized by aluminium and has a hcp-structure, while the $\beta$-phase is stabilized by vanadium, consisting of a bcc-lattice [28]. This batch of material was produced using the triple vacuum arc remelting (VAR) method. After production, a heat treatment at 750 $^\circ$C for 90 min was performed followed by air cooling. The chemical composition as specified from the supplier is given in Table 4 and the tensile properties (minimum values) in Table 5.

The material batch of Ck45 (C45E) was produced in an electric shaft furnace and afterwards rolled into cylindric form. The chemical composition as specified by the supplier is shown in Table 4, and the tensile properties of the materials in Table 5. A Jominy test was performed by the supplier, and the results are shown in Table 6. The material exhibits a high hardenability at the outer surface where the hardness is up to 59 HRC which indicates a martensitic microstructure and decreases to 19 HRC with increasing distance from the surface, indicating a ferritic-perlitic microstructure.

**Table 4:** Supplier information on the chemical composition of the material batches of Ti6Al4V and Ck45

Material	Fe	C	N	H	O	Y	Al	V
Ti6Al4V	0.111	0.025	0.020	0.003	0.15	$<\!0.005$	6.12	4.11
Ck45	Balance	0.445	–	–	–	–	0.01	0.002
	Ti	Mn	Si	P	S	Cr	Ni	Mo
Ti6Al4V	Balance	–	–	–	–	–	–	–
Ck45	0.01	0.76	0.24	0.018	0.02	0.18	0.05	0.01
	Cu	Sn	Nb	B	Residuals			
					each	total		
Ti6Al4V	–	–	–	–	$<\!0.1$	$<\!0.4$		
Ck45	0.15	0.006	0.001	0.000	–	–

**Table 5:** Supplier information on tensile test results of the respective Ti6Al4V and Ck45 material batch

Dimension	Ti6Al4V	Ck45
Tensile strength $R_m (MPa)$	952	671
Yield strength $R_{p0.2}(MPa)$	869	420
Elongation at break (%)	16.5 (4*D*[29])	22.2 ($A_5$[30])
Reduction of area (%)	39	–
Hardness test (HRC)	30.0	–

#### Sample preparation

**Fig. 13:**
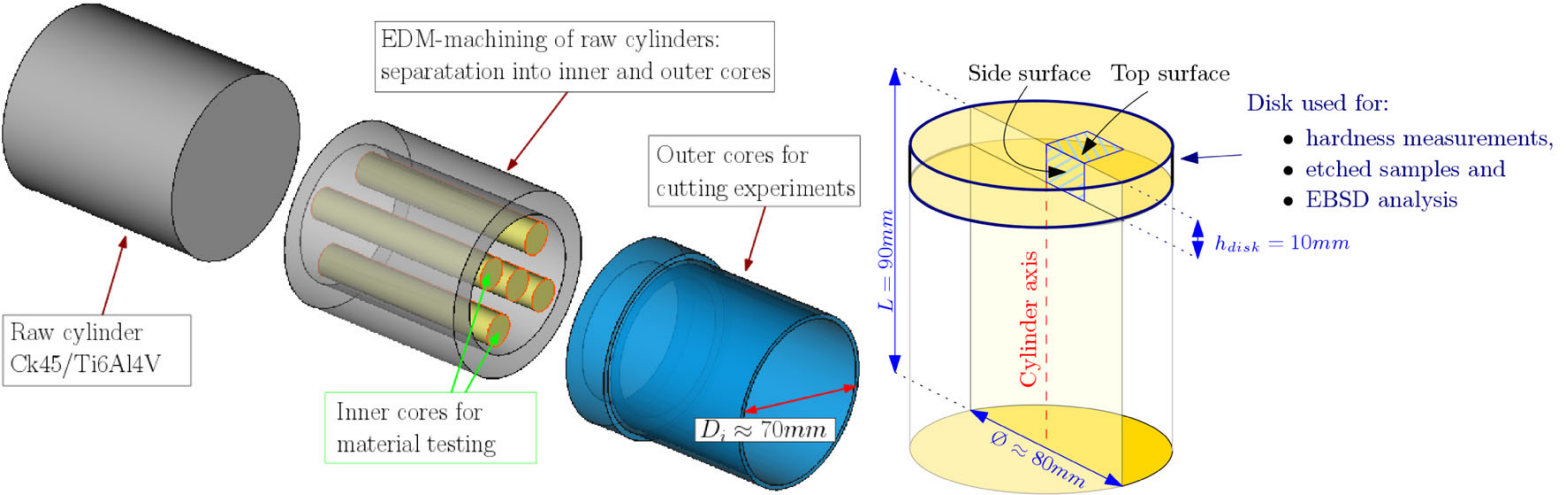
Raw material usage for material tests and orthogonal cutting tests (left) and sample orientations for hardness measurements, etched samples, and EBSD analysis with surface denominations (right)

Deep bores are inserted into the raw cylinders using EDM drilling. A smaller cylinder of 60 mm diameter is cut out via wire EDM and used to manufacture the test specimens for tensile testing (see Section 2.4.4). The outer cylinders are used for orthogonal cutting experiments. A disk with a height of $h_{disk}$ = 10 mm is sliced off of one cylinder of each material using wire EDM and used to determine the radial hardness profile. The disks are ground and polished prior to the hardness measurements. After the hardness measurements, the same disks are used to prepare etched samples from the top and side surfaces for microstructural investigations and EBSD analysis. An overview of the material usage is shown in Fig. 13.

#### Vickers hardness measurement

**Table 6:** Supplier information on Jominy test results of this material batch of Ck45

(mm)	1	2	3	4	5	6	7	8	9	10	11	13	15	20	25	30
HRC	59	57	54	46	37	34	30	29	28	27	26	25	24	23	21	19

**Fig. 14:**
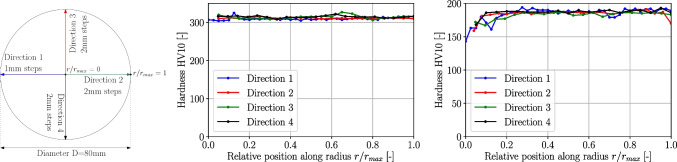
Hardness measurement directions (left) and results for Ti6Al4V (middle) and Ck45 (right)

**Fig. 15:**
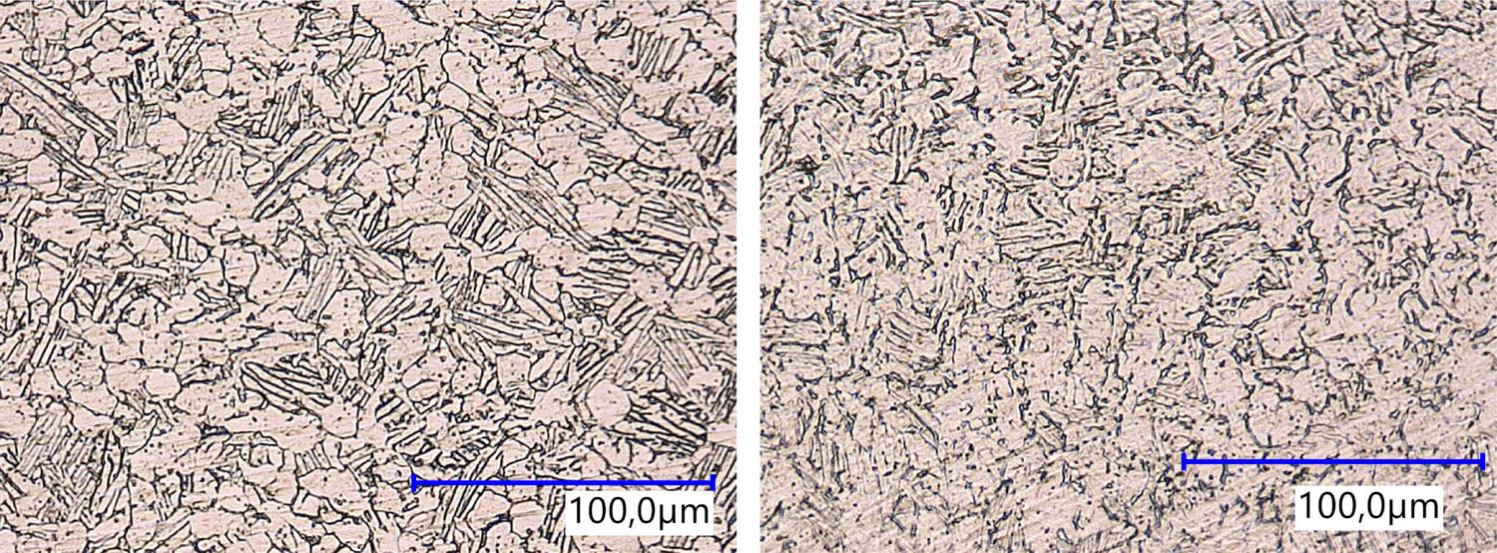
Microstructure of Ti6Al4V: the top surface (left) and the side surface (right) show a uniform grain structure

**Fig. 16:**
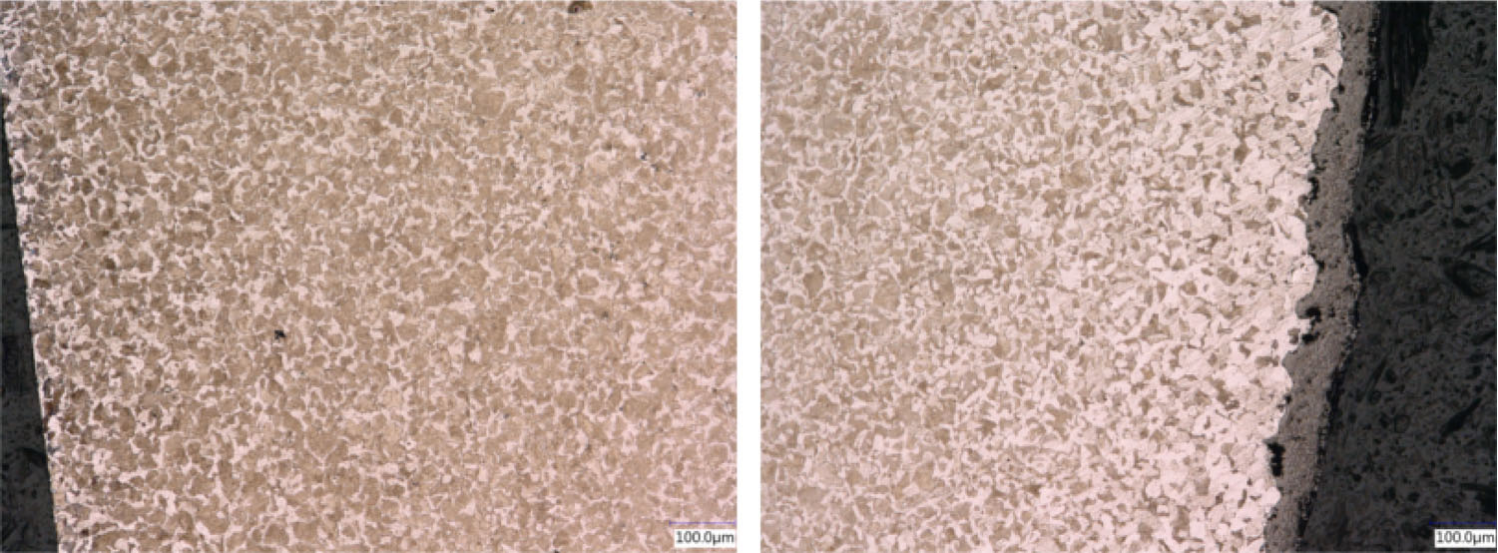
Microstructure of Ck45 steel: top surface at the disk center (left) showing a uniform grain structure and at outer radius (right) showing decarburation and mill scale

Vickers hardness measurements HV10 are conducted for Ck45 and Ti6Al4V. The measurements are performed on the top faces of each cylinder along four directions of the samples where in one direction a 1 mm stepping and in the others a 2 mm stepping was used. The hardness measurement directions and the results of the hardness measurement for both materials are shown in Fig. 14. The Ck45 has an average hardness of 184 HV10 but shows a hardness reduction down to 143 HV10 at the disk center within a radius of around 6 mm. Towards the outer radius, the hardness is constant except for the very last measurement point in direction 2 which shows a slight drop in hardness. The hardness distribution of the Ti6Al4V sample is almost constant in all directions and radial positions with the exception of some spots. The average hardness is 312 HV10.

#### Microstructure

The microstructure of the two materials is investigated by optical analysis of the etched surface and EBSD analysis. This investigation should indicate possible irregularities of the grain structure or anisotropy in the material which could lead to different requirements for the constitutive model to be used in numerical simulations of the cutting experiments.

Etched samples are prepared for microstructural analyses of the top and side surfaces. The Ti6Al4V samples are etched with Kroll. The microstructure of the top and side surfaces are shown in Fig. 15. Due to the heat treatment, both show a uniform microstructure without any salience.

**Fig. 17:**
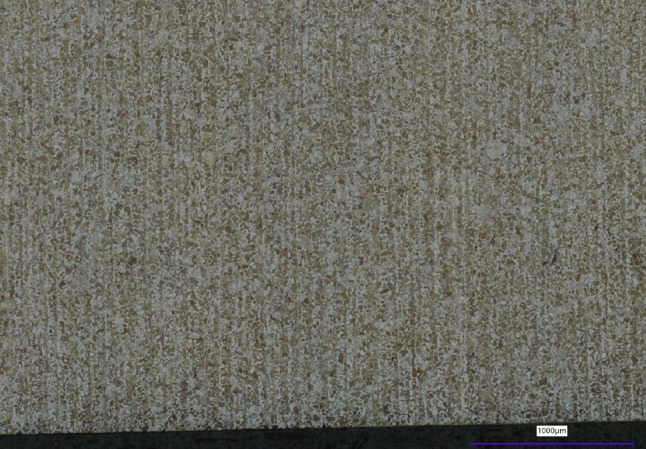
Microstructure of Ck45 steel: side surface showing a columnar structure

The Ck45 is etched with Nital. Figure 16 shows the top surface. A ferritic-perlitic microstructure is visible. Towards the outer surface, decarburations and mill scales can be seen. At the disk center, the microstructure is uniform. The side surface microstructure revealed a columnar structure along the cylinder axis (see Fig. 17). This is likely to have been induced by rolling during the manufacturing process.

**Table 7:** EBSD measurement sizes of the four specimen

Specimen	Surface	Sample size $(\mu m^2)$	Figures
Ti6Al4V	Side	$443 \times 347$	18, 19, 20
Ti6Al4V	Top	$331 \times 428$	
Ck45	Side	$545 \times 428$	21, 22
Ck45	Top	$709 \times 556$	

**Fig. 18:**
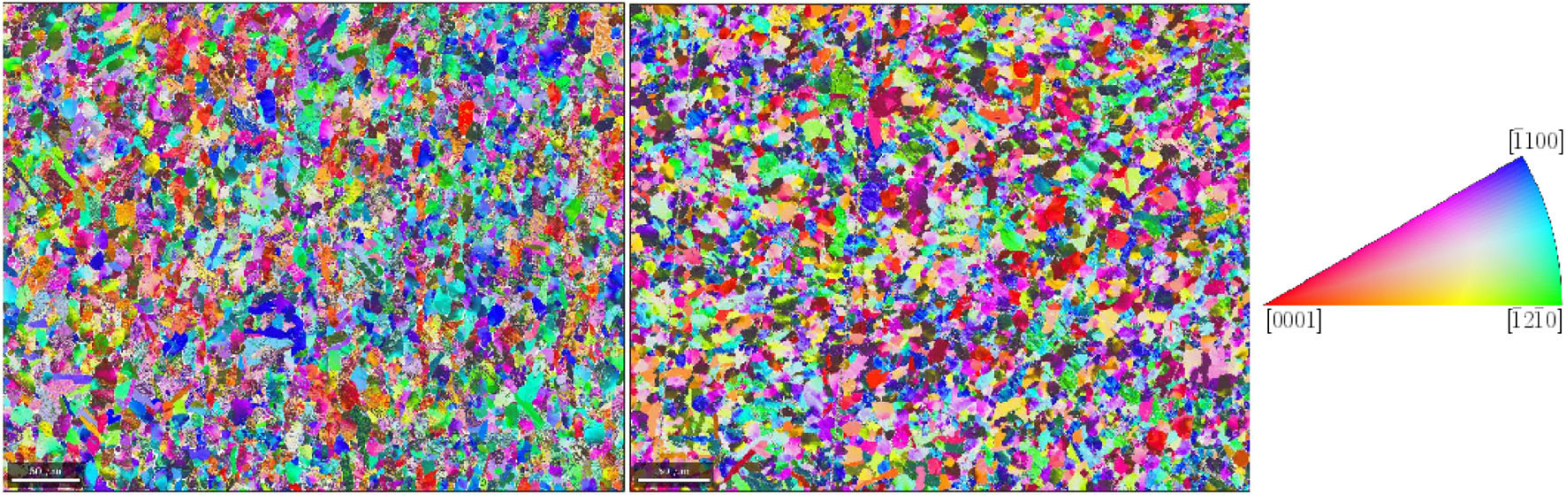
EBSD of Ti6Al4V: side (left) and top (middle) surface with crystal orientations (right)

**Fig. 19:**
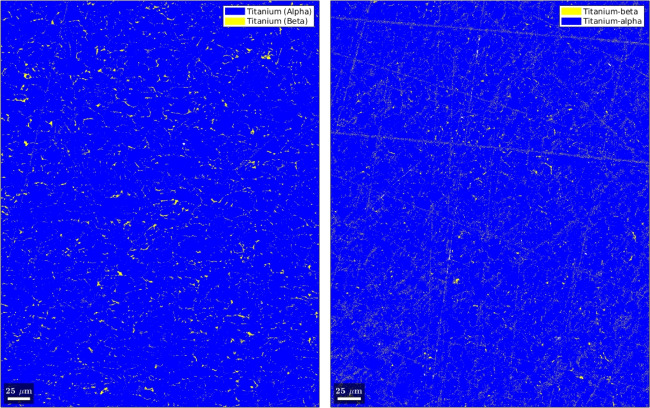
EBSD of Ti6Al4V: $\alpha$ / $\beta$-phase distribution in the side (left) and top (right) surface

**Fig. 20:**
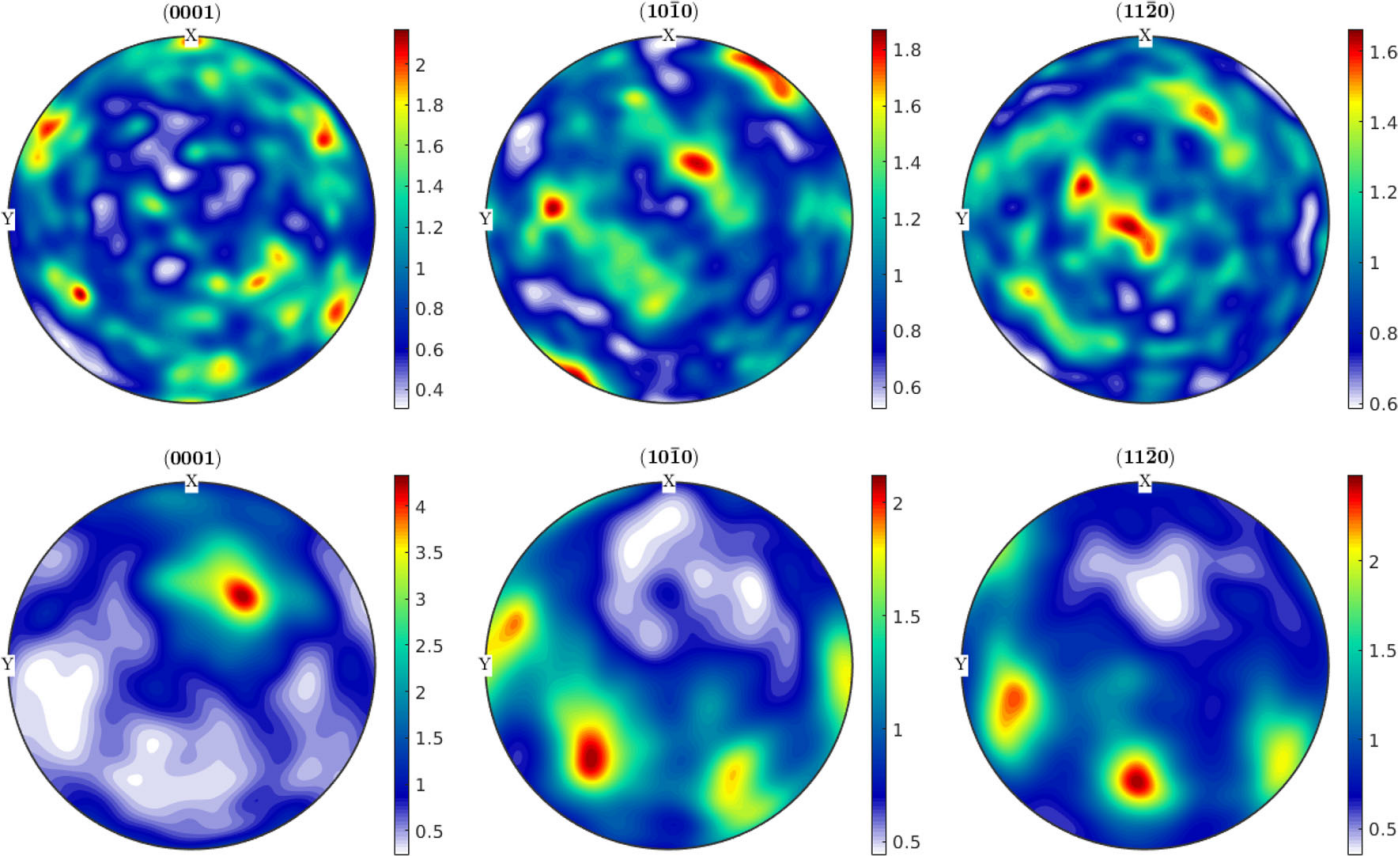
Pole figures 0001, $10\bar{1}0$, and $11\bar{2}0$ of Ti6Al4V: top (top row) and side (bottom row) surface of the $\alpha$-phase

**Fig. 21:**
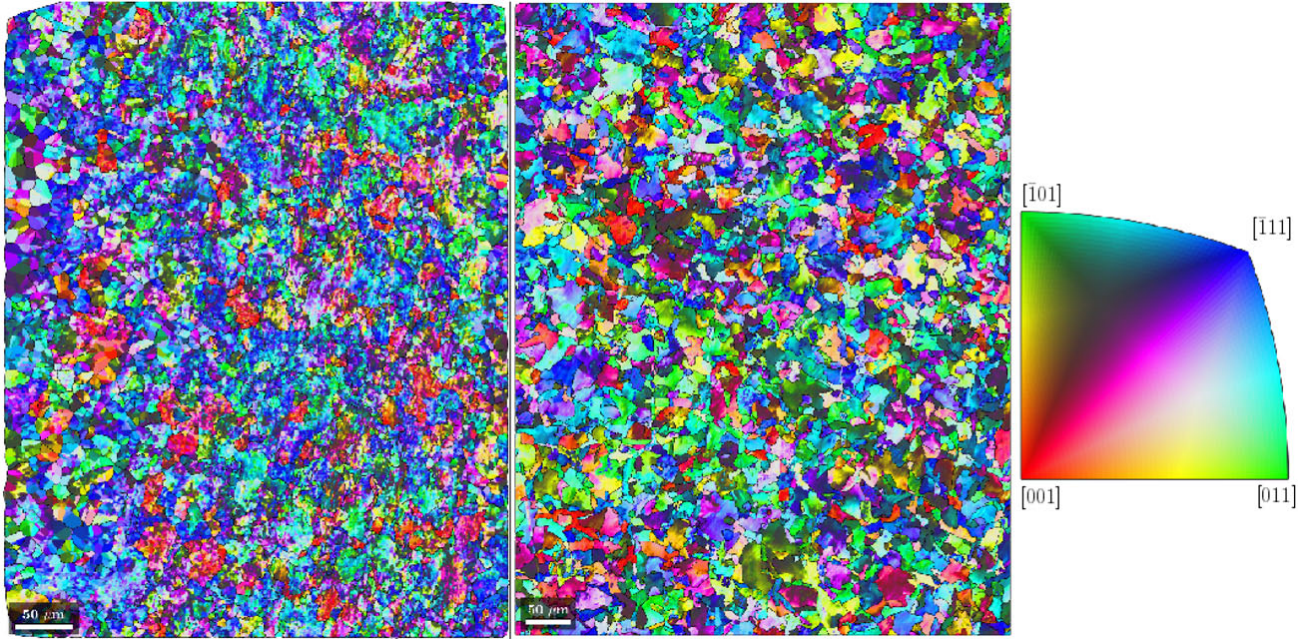
EBSD of Ck45: side (left) and top (middle) surface with crystal orientations (right)

**Fig. 22:**
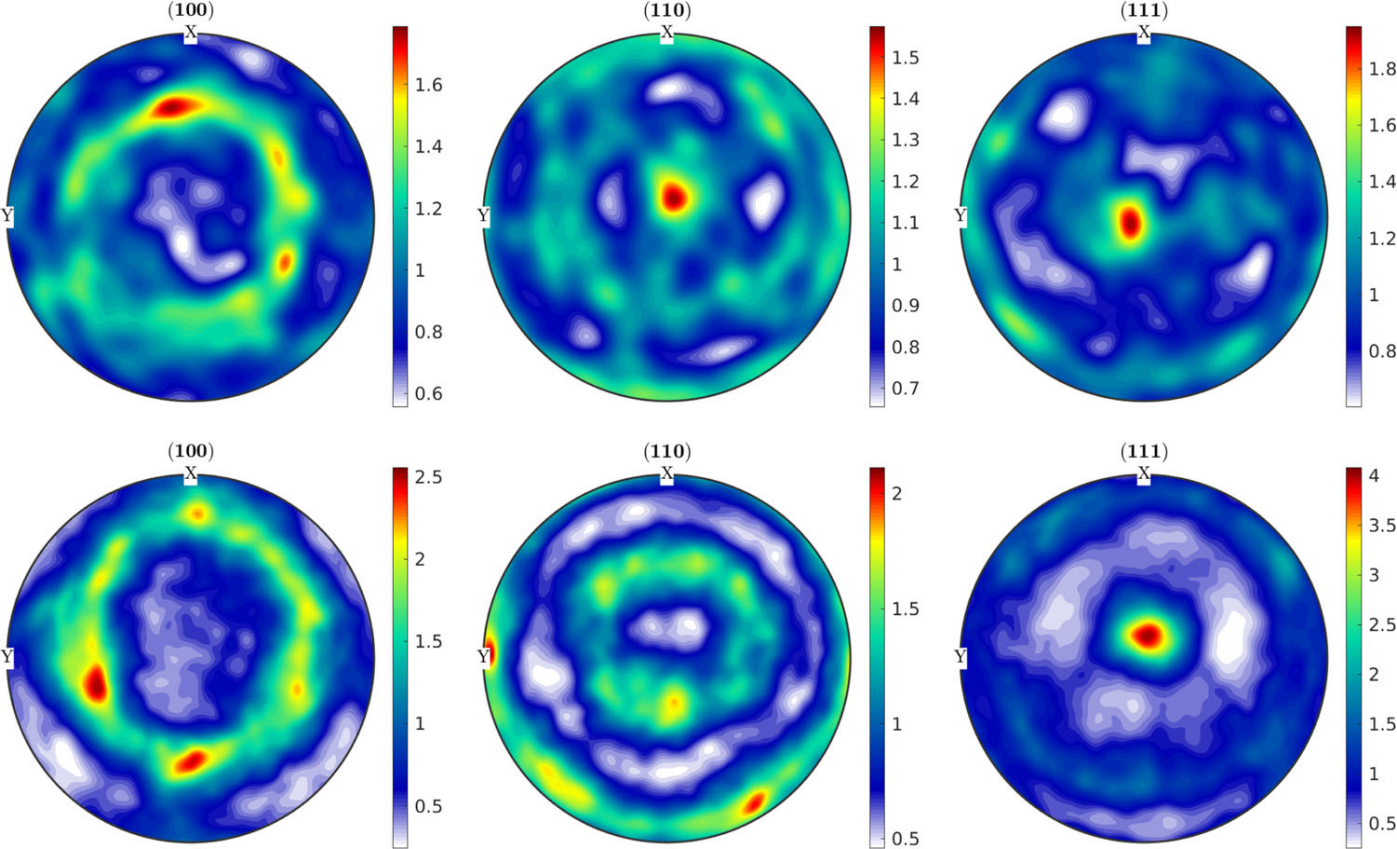
Pole figures 100, 110, and 111 of Ck45: top (top row) and side (bottom row) surface

The crystallographic orientations in the grains and the textures are measured with EBSD for the top and side surfaces of the Ck45 and Ti6Al4V material. The measured areas of the samples are given in Table 7. The EBSD analysis of Ti6Al4V reveals a slightly different ratio of $\alpha$- and $\beta$-phase for top and side surfaces. The average grain diameters and average aspect ratios of the grains are similar for the side and top surfaces. The results, together with grain sizes and aspect ratios, are given in Table 8 for Ti6Al4V and Ck45. The grain orientations of the $\alpha$-phases of Ti6Al4V are shown for the side and top surface in Fig. 18, the corresponding distributions of $\alpha$ and $\beta$-phases in Fig. 19. The pole figures of the $\alpha$-phases are given with Fig. 20 for side and top surfaces, respectively. The pole figures reveal a slightly stronger texture on the side surface than on the top surface. The grain orientations of Ck45 are shown for the side and top surfaces in Fig. 21 and the pole figures in Fig. 22. Similar to Ti6Al4V, the side surface shows a stronger texture than the top surface which may have been induced by the rolling process during manufacturing.

#### Tensile tests

**Fig. 23:**
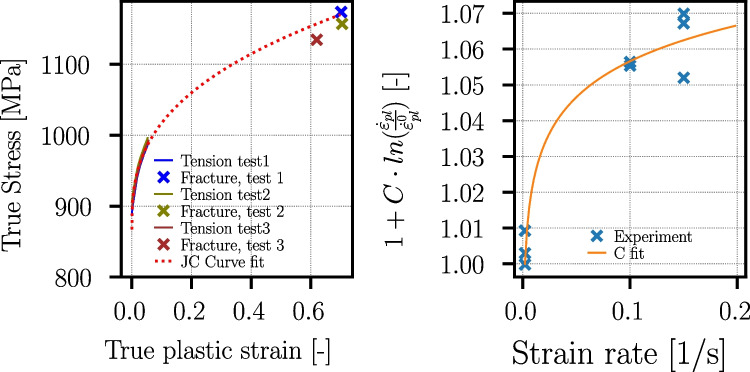
Ti6Al4V: measured stress–strain curves with fracture stresses and strains for the three tensile tests at quasi-static conditions ($\dot{\varepsilon }_{pl}\!=\!0.002s^{-1}$) and fit of the Johnson-Cook parameters *A*, *B*, and *n* (left) and fit of the Johnson-Cook strain rate sensitivity parameter *C* (right)

**Fig. 24:**
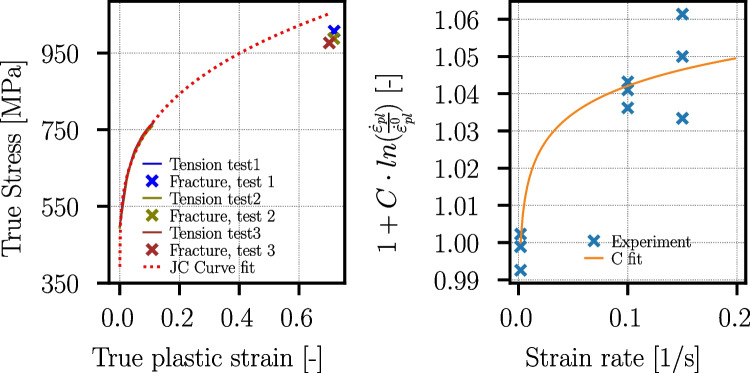
Ck45: measured stress–strain curves with fracture stresses and strains for the three tensile tests at quasi-static conditions ($\dot{\varepsilon }_{pl}\!=\!0.002s^{-1}$) and fit of the Johnson-Cook parameters *A*, *B*, and *n* (left) and fit of the Johnson-Cook strain rate sensitivity parameter *C* (right). Note that the Lüders band formation is cut off in the displayed experimental flow curve

In the following, tensile test results on this batch of materials are summarized from [24]. The tensile tests are performed for both materials, Ck45 and Ti6Al4V, at room temperature for three different strain rates ($\dot{\varepsilon }_{pl}={0.002}\,{\text {s}^{-1}},{0.1}{\text {s}^{-1}},{0.15}\,{\text {s}^{-1}}$) and each repeated three times. The tensile test specimens are produced according to DIN50125 [31] with form B and the dimensions B8x40 from the inner core of the cylinders (see Fig. 14). The tensile tests revealed yield strengths at quasi-static conditions in the order of $\sigma _y = {867}\,{\text {MPa}}$ for Ti6Al4V. For Ck45, Lüders band formation is observed, and the yield strength is in the order of $\sigma _y = {392}\,{\text {MPa}}$. The measured flow stress curves are used to fit material parameters for the work hardening and strain rate sensitivity part of the Johnson-Cook flow stress model [32]:

3 $$\begin{aligned} \begin{aligned} \sigma _y =&\underbrace{\left( A+B \cdot (\varepsilon _{pl})^n\right) }_{\text {work hardening}} \cdot \underbrace{\left( 1+C \cdot ln\left( \frac{\dot{\varepsilon }_{pl}}{\dot{\varepsilon }^0_{pl}}\right) \right) }_{\text {strain rate sensitivity}} \cdot \\&\underbrace{\left( 1- \left( \frac{T-T_{ref}}{T_f-T_{ref}} \right) ^m \right) }_{\text {thermal softening}} \end{aligned} \end{aligned}$$

where *A*, *B*, *C*, *m*, and *n* are material dependent parameters, $\varepsilon _{pl}$ is the equivalent plastic strain, $\dot{\varepsilon }_{pl}$ the plastic strain rate, *T* the temperature, $T_f$ the melting temperature, and $T_{ref}$ the reference temperature of the material tests. Tests at temperatures higher than room temperature are not carried out; therefore, the parameter *m* stays undetermined. The parameters *A*, *B*, *C*, and *n* are determined with the procedure in [33] using the measured true stress–strain curves and the fracture stresses and strains using the Bridgman correction [34]. The strain rate sensitivity parameter C is valid only at very low strain rates, as the tests are conducted in the strain rate range from ${0.002}\,{\text {s}^{-1}}$ to ${0.15}\,{\text {s}^{-1}}$. The obtained Johnson-Cook parameters are given in Table 9. The experimental and JC-approximated flow curves are given together with the fit of the strain rate sensitivities in Fig. 23 for Ti6Al4V and in Fig. 24 for Ck45.

**Table 8:** Ti6Al4V and Ck45: EBSD measurement results for side and top surfaces

Material	Ti6Al4V		Ck45	
Surface	Side	Top	Side	Top
Grain diameter, average $D_g (\mu m)$	9.9	9.1	17.8	14.3
Grain diameter, std.dev. $\mu _{D_g} (\mu m)$	4.2	3.2	13.5	9.3
Grain aspect ratio, average	1.8	1.7	1.8	1.8
Grain aspect ratio, std.dev	0.6	0.5	0.5	0.6
$\alpha$-phase vol. %	96.7	94.8	-	-
$\beta$-phase vol. %	3.3	5.2	-	-

**Table 9:** Ti6Al4V and Ck45: fit of the work hardening parameters *A*, *B*, and *n* and the strain rate sensitivity *C* of the JC flow stress model (3)

Material	*A*	*B*	*n*	$\dot{\varepsilon }_{pl}^{ref}$	*C*
	(MPa)	(MPa)	(-)	(1/*s*)	(-)
Ti6Al4V	867	344	0.361	0.002	0.0145
Ck45	392	735	0.304	0.002	0.0108

#### Thermophysical properties

Thermal and physical properties are not measured on the raw materials; therefore, typical values from the literature [35] are presented in Table 10.

**Table 10:** Ti6Al4V and Ck45: EBSD measurement results for side and top surface

Quantity	Symbol	Unit	Ti6Al4V	Ck45
Young’s modulus	*E*	$({\text {GPa}})$	110	205
Poisson’s ratio	$\nu$	$(-)$	0.342	0.29
Density	$\varrho$	$(kg/m^3)$	4430	7870
Thermal conductivity	$\lambda$	(*W*/*mK*)	7.44	50.7
Specific heat	$c_p$	(*J*/*kgK*)	553	486
Melting temperature	$T_m$	(*K*)	1877	1773

## Results

Tables 15 and 16 in the appendix list the process parameters for each experiment ID and indicate for which experiment chip thicknesses are measured and for which etched samples are documented. The complete cutting test results are provided for Ti6Al4V in Table 17 and for Ck45 in Table 18 in the C. The process forces $F_c$ and $F_f$ are normalized to a cutting width of $w=1mm$ and are given together with their respective standard deviations $\sigma _{F_c}$ and $\sigma _{F_f}$. The next two columns contain the averaged cutting edge radius $r_n$ and its standard deviation $\sigma _{r_n}$, followed by the average chip thickness $h_{avg}$ and its standard deviation $\sigma _{h_{avg}}$ (if more than one chip is evaluated), the cutting distance $l_{cut}$ and the temperature of the chip $T_{chip}$ derived from the tempering colour (only Ck45). Since in some cases, significant tool wear occurs at high cutting speeds and or high feed rates, the last two columns contain a statement about tool wear and the stability of the measured process forces. Experiments with unstable process forces or with high tool wear are not considered in the following evaluation.

**Fig. 25:**
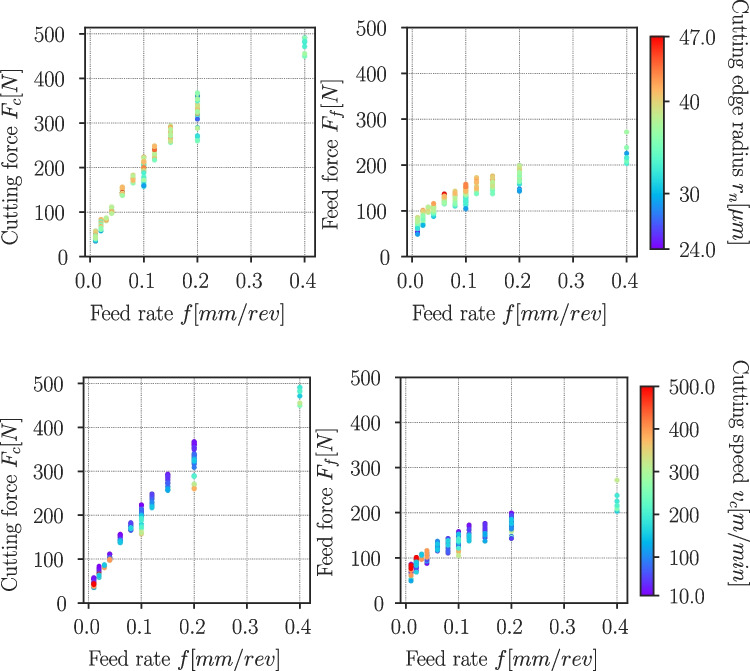
Process forces Ti6Al4V cutting experiments, cutting (left column) and feed force component (right column) depending on the feed rate *f*, the colour depicts the cutting edge radius $r_n$ (top row) and the cutting speed $v_c$ (bottom row) of the experiment

**Fig. 26:**
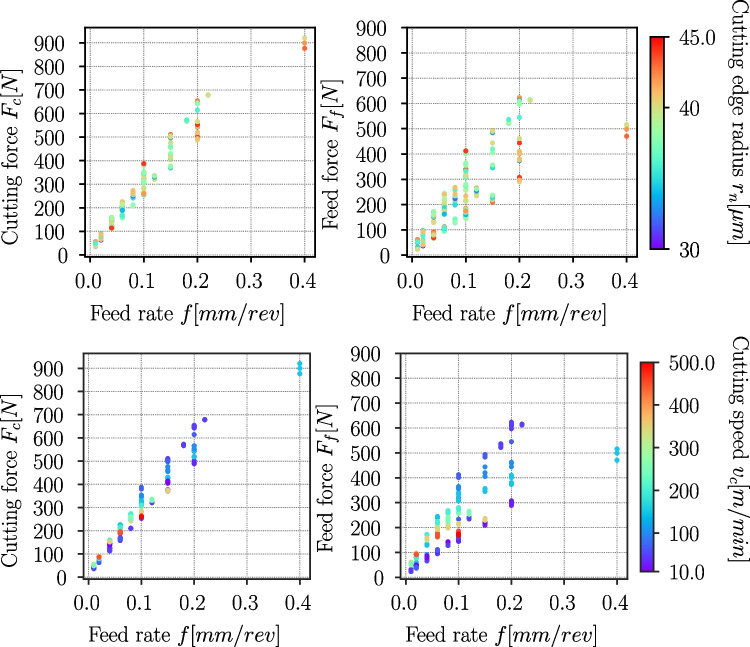
Process forces Ck45 cutting experiments, cutting (left column) and feed force component (right column) depending on the feed rate *f*, the colour depicts the cutting edge radius $r_n$ (top row) and the cutting speed $v_c$ (bottom row) of the experiment

### Process forces and specific cutting energy

The experimentally measured process forces are normalized to a cutting width of $b={1}\,{\text {mm}}$ and are displayed for Ti6Al4V and Ck45 in Figs. 25 and 26, respectively. The colour of the dots indicates the cutting edge radius $r_n$ or cutting speed $v_c$ of the respective experiment. For Ti6Al4V, cutting and feed force increase with increasing feed rate. The cutting forces $F_c$ tend to increase with increasing cutting edge radius $r_n$, but decrease towards higher cutting speed $v_c$. The feed force $F_f$ increases as well with increasing $r_n$, but it appears that increasing $v_c$ leads to higher forces below feed rates of around $f={0,1}\,{\text {mm/rev}}$.

The Ck45 exhibits a complex behaviour: the process forces increase as well with increasing feed rate, but dependencies on the cutting edge radius $r_n$ or cutting speed $v_c$ are not obvious. Instead, at a constant feed rate, the process forces first increase from very low to medium cutting speeds ($v_c \approx {100}\,{\text {m/min}}$) and then start to drop to similar low forces as with very low cutting speeds (see for example the feed forces at $f={0,1}\,{\text {mm/rev}}$ in Fig. 26).

**Fig. 27:**
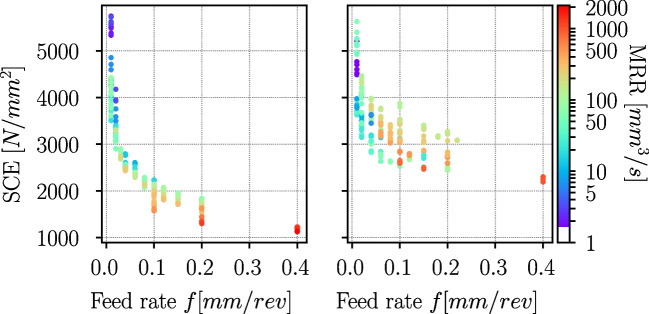
Specific cutting energy (SCE) versus feed rate *f* for Ck45 and Ti6Al4V. The colouring of the data points depicts the corresponding material removal rate (MRR)

With the measured cutting forces, the specific (volumetric) cutting energy (SCE) $e_c$ can be calculated according to [36]:

4 $$\begin{aligned} e_c = \frac{F_c v_c}{b h v_c} = \frac{F_c}{A_c} = k_c \end{aligned}$$

with $F_c$ being the cutting force, $v_c$ the cutting speed, *b* the cutting width, *h* the uncut chip thickness, $A_c$ the cross-sectional area of the uncut chip and $k_c$ the specific cutting pressure. The material removal rate (MRR) is defined as follows:

5 $$\begin{aligned} MRR = v_c b h = A_c v_c \end{aligned}$$

The computed SCE are displayed in Fig. 27 versus the feed rate. The colouring of the data points corresponds to the MRR. For both materials, the SCE decreases with increasing feed rate as well as MRR.

**Table 11:** Ti6Al4V: chip overview of selected experiments with the chip flow direction in all images from right to left

f	Scale	Cutting speed ${v_{c}} {(}m{/}min{)}$			
(*mm*/*rev*)	$100\mu m$	11	40	100	381
0.01					
0.1					
0.2					

Towards higher cutting speeds and higher feed rates, the chip segmentation becomes more pronounced

### Chip shapes

At least one chip sample of each combination of feed rate and cutting speed is embedded and etched. Cutting experiments for which etched images are available are provided in Tables 15 and 16 in Appendix 2, and they can be downloaded from the location provided in Table 13. In the following, a small selection is shown for Ti6Al4V and Ck45. For Ti6Al4V, chips in a feed rate range of $f=0.01mm/rev, 0.1mm/rev$ and 0.2*mm*/*rev* and at cutting speeds of $v_c=11, 40, 100$ and 381*m*/*min* are displayed in Table 11. All chips are continuous with a wavy or segmented chip type. The chip segmentation becomes more pronounced towards higher feed rate *f* and higher cutting speed $v_c$. None of the chips from the Ti6Al4V cutting experiments showed traces of the effect of built-up edges (BUE).

**Fig. 28:**
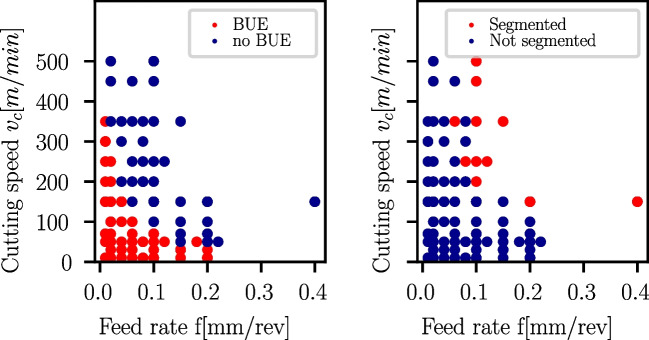
Process conditions in Ck45 machining at which built-up edge formation traces are found in the chips (left), and chip segmentations occur (right)

For Ck45, chips in a feed rate range of $f=0.02mm/rev, 0.06mm/rev$ and 0.1*mm*/*rev* and at cutting speeds of $v_c=10, 50, 150$ and 450*m*/*min* are displayed in Table 12. Ck45 chips show BUE traces in the chips up to $v_c=50m/min$ at all three feed rate levels, while at $v_c=150m/min$, only for $f=0.02mm$ BUE is visible, and for $v_c=450m/min$, no BUE is visible at all. At higher cutting speeds and higher feed rates of the Ck45 cutting experiments, the BUE-formation diminishes, while chip segmentations start to form. The BUE and chip segmentation behaviour versus cutting speed and feed rate is displayed in Fig. 28.

**Table 12:** Ck45: chip overview of selected experiments with the chip flow direction in all images from right to left

f	Scale	Cutting speed ${v_{c}} {(}m{/}min{)}$			
(*mm*/*rev*)	$100\mu m$	10	50	150	450
0.02					
0.06					
0.1					

Traces of BUE are encircled in blue. Towards higher cutting speed and higher feed rate, chip segmentation starts to form, while traces of BUE diminish and disappear

**Fig. 29:**
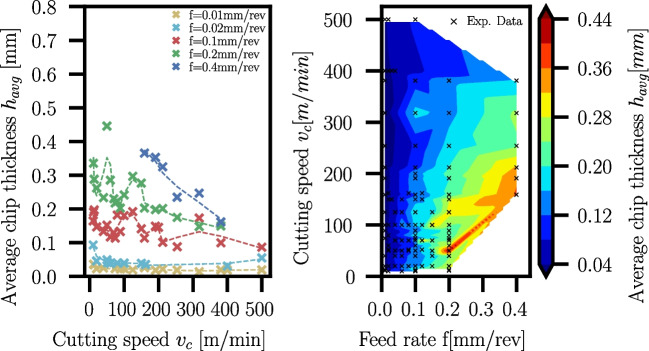
Measured average chip thickness of Ti6Al4V for different feed rates *f* and varying cutting speeds $v_c$. Evaluation for selected feed rates with smoothed trendlines (left) and contour plot with black dots used for the interpolation (right)

### Chip thickness

At least one chip of each combination of feed rate and cutting speed is embedded in Bakelite for the manual measurement of $h_{avg}$ using the method described in Section 2.3. The measured thicknesses are documented in Appendix 3 in Tables 17 and 18 for Ti6Al4V and Ck45, respectively. The average chip thicknesses scatter for Ti6Al4V and Ck45. For each chip, the averaged chip thickness was measured in several areas, and standard deviations $\sigma _{h_{avg}}$ were determined from them, which are additionally given in the result tables in the appendix. The scatter can occur due to various reasons:

- The manual measurement procedure of the chip area and the polygonal approximation of the unrolled chip length.
- Misalignments of chips in the embedding procedure because the chips are curled not only in feed direction but also due to the radius of the cutted cylinder.
- Low stiffness of thinner chips which could lead to skewing during the embedding process, and therefore, larger effective cross-sections can appear.

For different feed rates, averaged chip thicknesses have been evaluated as a function of cutting speed. Since the chip thickness measurements scatter for the reasons mentioned above, smoothed trendlines are created using the Savitzky-Golay filter [37] in SciPy [38]. In addition to the trendlines, contour plots of chip thicknesses are created over feed rates and cutting speeds, interpolating between available data points.

**Fig. 30:**
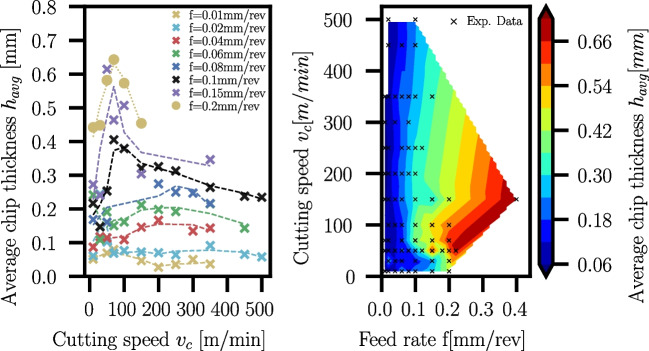
Measured average chip thickness of Ck45 for different feed rates *f* and varying cutting speeds $v_c$. Evaluation for selected feed rates with smoothed trendlines (left) and contour plot with black dots used for the interpolation (right)

**Fig. 31:**
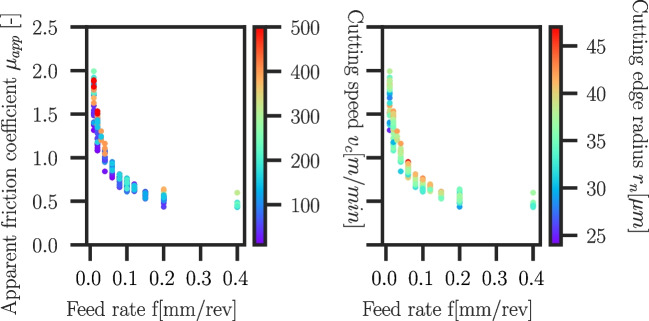
Apparent friction coefficients $\mu _{app}$ versus feed rate *f* for Ti6Al4V . The colour indicates the cutting speed $v_c$ (left) and the cutting edge radius $r_n$ (right). $\mu _{app}$ decreases with increasing feed rate *f* and decreasing cutting speed $v_c$

A trend of decreasing chip thicknesses towards an increase of the cutting speed $v_c$ at constant feed rate *f* is visible for both Ti6Al4V and Ck45 (see Figs. 29 and 30, respectively). In case of Ck45, an initial increase of the average chip thickness with increasing cutting speed for feed rates $f \ge {0,04}\,{\text {mm}}$ is seen. However, upon further increase of the cutting speed, the average chip thickness decreases again. The cutting speed at which the behaviour reverses is not the same for all feed rates: as the feed rate increases, the maximum of the average chip thickness moves towards smaller cutting speeds $v_c$.

### Friction coefficient

The measured process forces in the cutting experiments can be used to deduce friction coefficients. The simplest estimation is based on the ratio of tangential and normal forces exerted on the tool, also known as apparent friction coefficient $\mu _{app}$. It is given according to Merchant [21, 39] as follows:

6 $$\begin{aligned} \mu _{app} = \frac{F_f + F_c \cdot tan \gamma }{F_c - F_f \cdot tan \gamma } \end{aligned}$$

with $F_f$ and $F_c$ being the feed and cutting components of the process forces and $\gamma$ being the rake angle. The drawback of the apparent friction coefficient $\mu _{app}$ is the neglection of the cutting edge radius influence assuming an ideal sharp tool (perfect wedge). The apparent friction coefficients are shown for Ti6Al4V cutting experiments in Fig. 31. Towards higher feed rates, the apparent friction coefficient decreases to levels of $\mu _{app} \approx 0.4..0.6$. Especially at low feed rates, it can be seen that, as expected, a higher cutting edge radius leads to higher apparent coefficients of friction, but $\mu _{app}$ also increases with higher cutting speed.

**Fig. 32:**
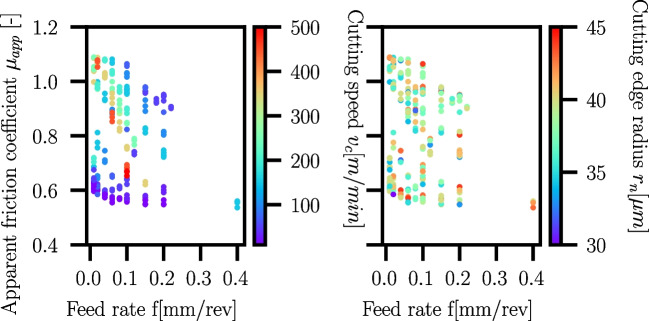
Apparent friction coefficients $\mu _{app}$ versus feed rate *f* for Ck45. The colour indicates the cutting speed $v_c$ (left) and the cutting edge radius $r_n$ (right). A dependency on the cutting edge radius $r_n$ is not visible, while for very low *f* and increasing cutting speed $v_c$, an increase in $\mu _{app}$ is visible which then drops upon a further increase in $v_c$. This behaviour is likely due to a change in BUE formation and chip segmentation

The apparent friction coefficients of the Ck45 cutting experiments are shown in Fig. 32. The cutting edge radius $r_n$ plays no obvious role, as lower and higher $r_n$ occur at all levels of $\mu _{app}$. For very low feed rates $f<0.05mm/rev$ and increasing cutting speed $v_c$, an increase in $\mu _{app}$ is visible. At higher cutting speeds, $\mu _{app}$ decreases with increasing feed rate.

**Fig. 33:**
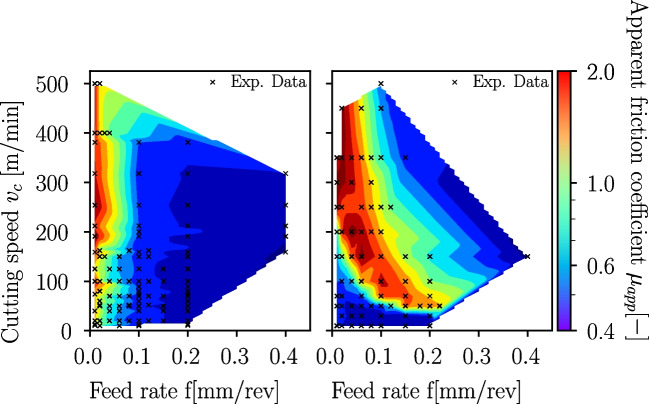
Apparent friction coefficients $\mu _{app}$ versus feed rate *f* and cutting speed $v_c$ for Ti6Al4V (left) and Ck45 (right). The colour indicates the magnitude of $\mu _{app}$ at the respective *f* and $v_c$. While for Ti6Al4V $\mu _{app}$ reduces with increasing *f*, Ck45 behaviour is more complex and shows a hyperbolic shape of the maximum values of $\mu _{app}$

Because the analysis of the apparent friction coefficients is not meaningful for Ck45, a contour plot is chosen instead, where the apparent friction coefficients are plotted versus cutting speed and feed rate. For comparison purposes, a similar plot is provided for Ti6Al4V as well in Fig. 33. While for Ti6Al4V the apparent coefficient is highest at small feed rates and almost independent from the cutting speed, a different behaviour can be seen for Ck45. The maximum values of $\mu _{app}$ are grouped along a hyperbolic curve. This hyperbola is located at the same combinations of *f* and $v_c$ where changes in the BUE-formation and chip segmentation are observed in Fig. 28.

**Fig. 34:**
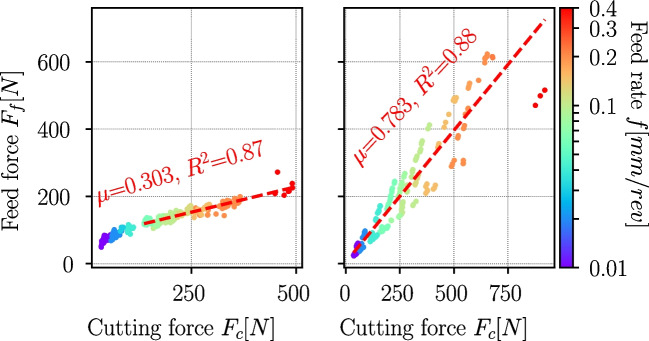
Friction coefficient $\mu _{fr}$ estimation based on Albrecht’s method for Ti6Al4V (left) and Ck45 (right). The estimated friction coefficient for Ck45 is very high and most likely inaccurate due to changes in BUE-formation and chip segmentation

A more elaborated approach for the determination of the friction coefficient from cutting experiments is described by Albrecht [19], considering the ploughing effect due to finite sharpness effect in the determination of the friction coefficient $\mu _{fr}$:

7 $$\begin{aligned} \mu _{fr} = \frac{(F_f-F_{pl,f}) + (F_c - F_{pl,c}) \cdot tan \gamma }{(F_c-F_{pl,c}) + (F_f - F_{pl,f}) \cdot tan \gamma } \end{aligned}$$

with $F_{pl,c}$ and $F_{pl,f}$ being the ploughing force components exerted in normal and feed direction. The friction coefficient $\mu _{fr}$ equals the slope in a $F_f-F_c$—plot where at higher feed rates ploughing effects diminish since the ratio of the cutting edge radius $r_n$ to the feed rate *f* becomes very small. Using the measured process forces of all Ti6Al4Vexperiments, the trend in Fig. 34 becomes visible from which the friction coefficient can be estimated with Albrecht’s method as $\mu _{fr}=0.3$.

**Fig. 35:**
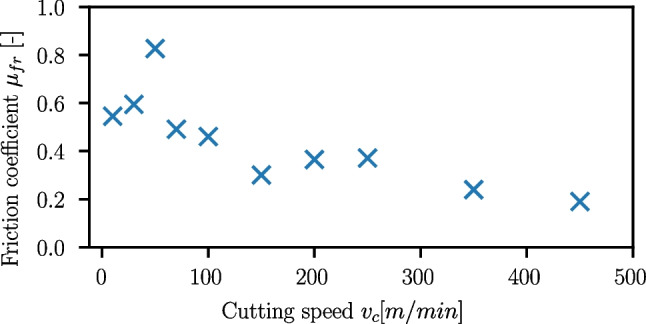
Ck45 friction coefficients determined with Albrecht’s approach showing a reduction with increasing cutting speed

However, for Ck45 the same method leads to a significantly higher friction coefficient of about $\mu _{fr}=0.78$, which is why cutting speed-dependent friction coefficients were evaluated instead and are shown Fig. 35.

From lower cutting speeds, the friction coefficient increases from $\mu _{fr}=0.545$ to a maximum of $\mu _{fr}=0.827$ at $v_c=50m/min$ and from there continuously drops to $\mu _{fr}=0.19$ at $v_c=450m/min$. The trend of the friction coefficients at cutting speeds beyond $v_c=50m/min$ is similar to the ones reported in [12] and in [40]. The reason for the observed trend is possibly linked to changes in BUE-formation and chip segmentation as discussed in Section 3.2. Detailed figures for each cutting speed can be found in Appendix 1.

It is emphasized that a more credible deduction of friction coefficients requires cutting tests with specially prepared cutting edge radii $r_n$ as for example used in [10]. Alternatively, an in-process tribometer can be used for the determination of the friction coefficient [41] directly on the fresh-cut surface.

### Tempering colours

**Fig. 36:**
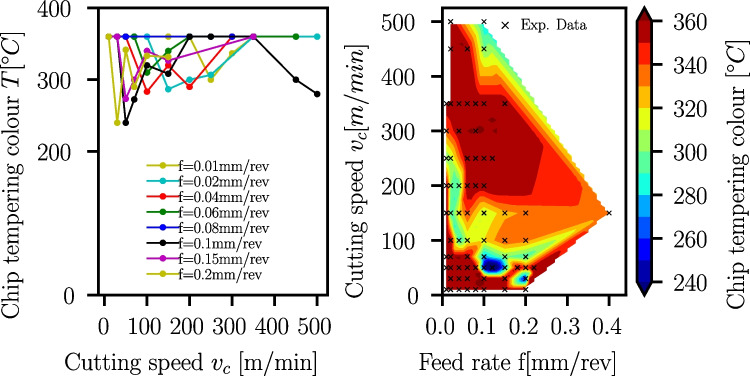
Estimated Ck45 chip temperatures based on tempering colours for different feed rates *f* and varying cuttings speeds $v_c$. Evaluation for selected feed rates (left) and interpolated contour plot (right) with black dots denoting the experimental data points which have been used for the interpolation

During the cutting, the temperature in the chips increases due to plastic dissipation in the primary shear zone and friction in the secondary shear zone. In the case of the Ck45, the temperature increase leads to visible tempering colours in the chips, which can be matched to tabulated values [42]. The tempering colours can be used to estimate the occuring chip temperatures in a range between $T_{min}=200^\circ C$ and $T_{max}=360^\circ C$. All Ck45 chips are manually analyzed, and the tempering colour dependence on the cutting speed is shown for selected feed rates together with interpolated contour plots in Fig. 36. At low cutting speeds, temperatures are in the order of the maximum temperature $T_{max}=360^\circ C$. With increasing cutting speed, the temperatures first reduce to levels below $T=300^\circ C$ between $v_c=100..200m/min$. In cutting speed ranges of $v_c \approx 200..450m/min$, the temperatures increase to $T_{max}\approx 360^\circ$ followed by a temperature reduction when the cutting speed is increased beyond $v_c \approx 450m/min$. A possible explanation of this effect is a change in the chip formation mechanism (see Section 3.2 and Fig. 28). This effect could be maybe induced by DSA, where at higher temperatures, the yield strength is increasing.

### Tool wear

The tool wear depends on the feed rate, cutting speed, and the amount of material removed at these parameters. For each cutting experiment, the tool wear is visually inspected and qualitatively classified into three categories:

- Low (L): no traces of crater wear or wear-land wear
- Medium (M): beginning crater and wear-land wear
- High (H): heavy crater and/or wear-land wear, cutting edge worn

**Fig. 37:**
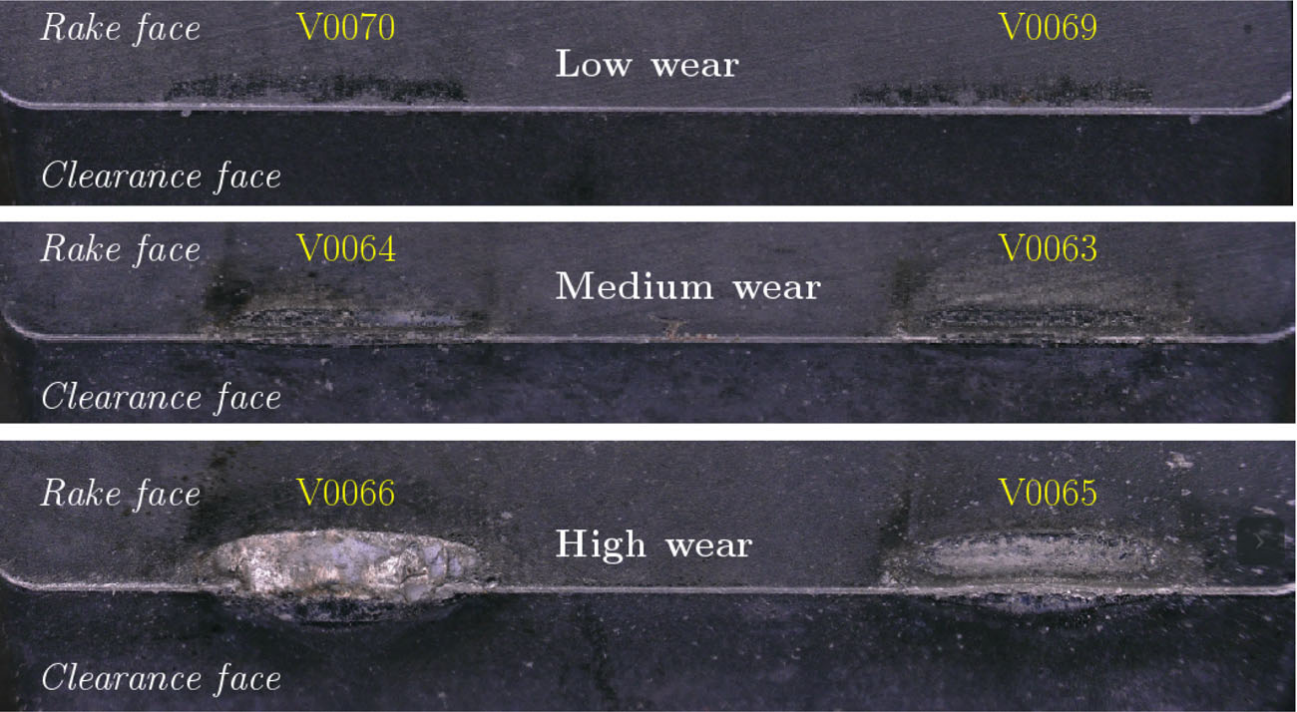
Wear classification of the cutter inserts: examples for low (top), medium (middle), and high (bottom) wear. The text in yellow colour depicts the corresponding experiment number

Examples of different tool wear classifications are shown in Fig. 37. The tool wear classification is given for each experiment in the appendix in Tables 17 and 18 for Ti6Al4V and Ck45, respectively.

**Fig. 38:**
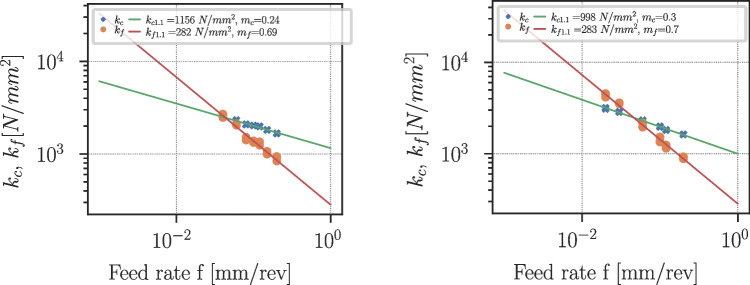
Kienzle coefficient determination for Ti6Al4V at cutting speeds of $v_c=70m/min$ (left) and $v_c=150m/min$ (right)

### Kienzle coefficients

The Kienzle-Victor process force model [43] is an empirical equation that relates the cutting force $F_c$ to the cut width *b*, uncut chip thickness *h*, and a factor $k_c$ which is the cutting force per unit area:

8 $$\begin{aligned} F_c = k_c \cdot a_p \cdot f = k_c \cdot b \cdot h \end{aligned}$$

All effects of the process conditions like cutting speed, tool geometry, and workpiece and tool material are contained in $k_c$. It was found by Kienzle that the cut width *b* scales linearly with the cutting force while the uncut chip thickness scales with a power law, where $k_{c1.1}$ is the specific cutting force at $b=h={1}\,{\text {mm}}$:

9 $$\begin{aligned} F_c = b \cdot k_{c1.1} \cdot h^{1-m_c} \end{aligned}$$

The model can be extended to feed and passive force as well [44]:

10 $$\begin{aligned} F_f= &   b \cdot k_{f1.1} \cdot h^{1-m_f} \end{aligned}$$

11 $$\begin{aligned} F_p= &   b \cdot k_{p1.1} \cdot h^{1-m_p} \end{aligned}$$

**Fig. 39:**
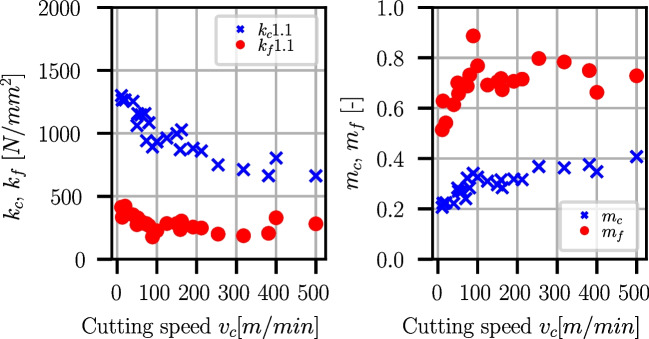
Kienzle coefficients for Ti6Al4V and various cutting speeds $v_c$

**Fig. 40:**
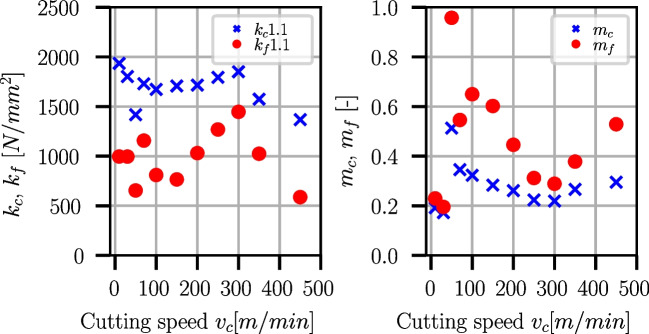
Kienzle coefficients for Ck45 and various cutting speeds $v_c$

In quasi-orthogonal cutting, the passive force component is $F_p \approx 0$, and therefore, Eqs. 9 and 10 are used to determine the Kienzle coefficients $k_{c1.1}(v_c), k_{f1.1}(v_c)$ and the respective exponents $m_c(v_c), m_f(v_c)$ at different cutting speeds for Ti6Al4V and Ck45. For this purpose, $k_c$ and $k_f$ are computed from the process forces and are extrapolated to $f=1mm$ for each cutting speed. Examples of such extrapolations are shown in Fig. 38 for two different cutting speeds.

Figure 39 shows the dependencies of the Kienzle coefficients on the cutting speed for Ti6Al4V. The corresponding values are supplied in Table 19 in Appendix 4. Towards higher cutting speeds, the specific cutting and feed force coefficients reduce, while the exponents $m_c, m_f$ increase.

For Ck45, the specific cutting and feed force coefficients slightly reduce until cutting speeds of $v_c = 150m/min$, followed by an increase towards $v_c = 300m/min$ and reduce from thereon strongly. The increase around $v_c = 300m/min$ is possibly induced by the dynamic strain aging (DSA) phenomenon [12, 14, 45]. Figure 40 shows the dependencies of the Kienzle coefficients on the cutting speed for Ck45. The corresponding values are supplied in Table 20 in Appendix 4.

### Data storage

The data is stored in the pCloud and contains process force measurement data, cutting edge radii scans, pictures of chip geometries, and etched chips (see the respective links in Table 13).

## Conclusions

Large-scale dry cutting experiments were conducted on Ti6Al4V and Ck45, with pre-test material characterization including hardness, microstructure, and tensile tests. The Johnson-Cook flow stress model coefficients were derived from these tests for the work hardening and strain rate sensitivity.

Cutting experiments used pristine cutting edges to minimize wear effects, and each edge’s radius was measured to account for its influence on process forces. For Ti6Al4V, the cutting edge radius affected process forces, with visible ploughing effects at lower feed rates. The friction coefficient was estimated at about 0.3, with chips showing continuous or wavy shapes and reduced thicknesses at higher speeds. Both cutting and feed forces decreased with increased cutting speed.

**Table 13:** Data storage of the quasi-orthogonal cutting experiments

Data	Link	Comment
Cutting test data	pCloud	All cutting test data
Process forces	pCloud	Format: time (s), $F_p (N)$, $F_f (N)$, $F_c (N)$
Cutting edge	pCloud	New inserts, informations in
scans		CutPositionsOnCuttingEdge.xls
Cutting edges	pCloud	
after tests		
Chip images	pCloud	Overview
Chip images	pCloud	Microscope images
(polished)		before etching
Chip images	pCloud	Microscope images with
(etched)		chip microstructures

For Ck45, the relationship between cutting edge radius, feed rate, and cutting speed was less clear. A contour plot indicated that the maximum apparent friction coefficient occurred at high speeds and low feed rates and shifted towards lower cutting speed to higher feed rates. Built-up edge formation and chip segmentation were associated with these friction coefficients. The specific cutting and feed forces decreased initially with cutting speed but increased beyond ${300}\,{\text {m min}^{-1}}$, with similar trends in chip thickness reduction. Complex cutting behaviour in Ck45 is likely due to dynamic strain aging effects. DSA effects are probably also responsible for reduced temperatures estimated from chip tempering colours in the area of the highest apparent friction coefficients. However, differences in the tempering colours could only be evaluated for a small temperature range, as this method cannot make any statements for temperatures below 200 $^\circ$C and above 360 $^\circ$C.

Friction coefficients were estimated but could be more accurately determined using specially ground cutting edges to reduce the scatter in the force measurements. Another possibility would be the use of an in-process tribometer. For more precise information on the process temperatures, it is advisable to record temperature curves using a pyrometer or IR camera. Measurement of chip thicknesses was challenging due to manual methods, chip embedding issues, and partial three-dimensional chip curvature. A planing setup could better approximate orthogonal cutting, though it limits maximum cutting speeds to around ${30}{\text {m min}^{-1}}$. The chip curling radii were not analyzed due to their variability during embedding.

## Supplementary Information

Below is the link to the electronic supplementary material.

Supplementary file 1 (xlsx 511 KB)

## Figures and Tables

**Fig. 41:**
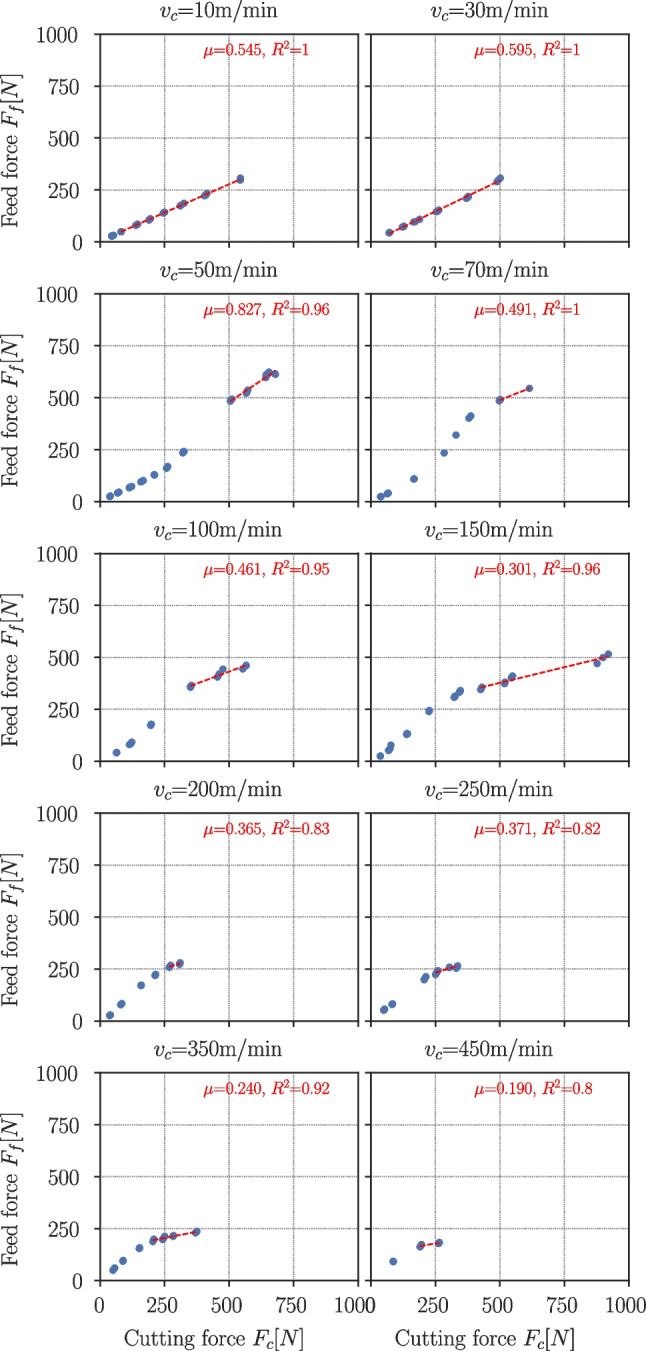
Friction coefficient $\mu _{fr}$ estimation based on Albrecht’s method [19] for Ck45 at different cutting speeds

## Data Availability

All data generated or analyzed during this study are included in this published article or a link to a cloud storage location is provided.

## Appendix 1. Friction coefficients for Ck45

The cutting speed dependent friction coefficients evaluated using Albrecht’s method [19] are given in Table 14, and the $F_c$ - $F_f$ diagrams for each cutting speed are shown in Fig. 41.

**Table 14:** Friction coefficients for Ck45 determined at different cutting speeds with Albrecht’s method

Cutting speed	$v_c$	$(m\!/\!min)$	10	30	50	70	100
Friction coefficient	$\mu _{fr}$	$(-)$	0.545	0.594	0.827	0.491	0.461
Cutting speed	$v_c$	$(m\!/\!min)$	150	200	250	350	450
Friction coefficient	$\mu _{fr}$	$(-)$	0.301	0.365	0.371	0.240	0.190

## Appendix 2. Test overview

Tables 15 and 16 contain the cutting experiment numbers sorted according to feed rate (columns) and cutting speed (rows) for Ti6Al4V and Ck45, respectively. The cutting experiment numbers can be used to find more information of the respective experiment in Tables 17 and 18. The colouring of the experiment number indicates the following:

- black: no chip thickness measurement available
- : chip thickness measurement available
- : chip thickness measurement and etched chip picture available

**Table 15:** Ti6Al4V: overview of process parameter combinations and according test numbers

**Table 16:** Ck45: overview of process parameter combinations and according test numbers

## Appendix 3. Experimental results

The complete cutting test results are provided for Ti6Al4V (V0001-V0068, V0301-V0520) in Table 17 and for Ck45 (V0069-V0300) in Table 18 below. Blue coloured experiment numbers contain a link to a high speed camera video of that test. The process forces $F_c$ and $F_f$ are normalized to a cutting width of $w=1mm$ and are given together with their respective standard deviations $\sigma _{F_c}$ and $\sigma _{F_f}$. The next two columns contain the averaged cutting edge radius $r_n$ and its standard deviation $\sigma _{r_n}$, followed by the average chip thickness $h_{avg}$ and its standard deviation $\sigma _{h_{avg}}$ (if more than one chip is evaluated), the cutting distance $l_{cut}$ and the temperature of the chip $T_{chip}$ derived from the tempering colour (only Ck45). The last two columns contain a qualitative statement of the tool wear and the process force measurement.

Tests labelled with  are without any objections. Tests labelled  have to be treated with care as such experiments should be repeated with a longer cutting distance in order for the process forces to reach a stable value. If however the standard deviations in the process forces of such tests are small, they still can be considered as valid. Results from tests labelled  or  are not trustworthy while tests labelled with  ran into the amplifier limits and tests marked  are most likely of insufficient quality and therefore invalid for further use. Nonetheless, invalid experiments are listed in the result tables for two reasons. First, critical process parameters are known then. Second, the generated chips can be still used for evaluation at these process conditions.

**Table 17:** Experimental results Ti6Al4V orthogonal cutting tests

Test	$v_c$	*f*	$F_c$	$\sigma _{F_c}$	$F_f$	$\sigma _{F_f}$	$r_n$	$\sigma _{r_n}$	$h_{avg}$	$\sigma _{h_{avg}}$	$l_{cut}$	$T_{chip}$	Wear	Force
	$(m\!/\!min)$	(*mm*/*rev*)	(*N*)	(*N*)	(*N*)	(*N*)	$(\mu m)$	$(\mu m)$	$(\mu m)$	$(\mu m)$	(*mm*)	(*K*)		measurement
V0001	10.5	0.01	55.7	2.1	83.8	4.9	37.1	2.0	34.4	9.1	2024	-	L	
V0002	12.6	0.01	55.2	1.8	76.4	4.2	32.0	2.4	38.3	8.3	2024	-	L	
V0003	10.5	0.01	57.4	1.8	80.7	4.6	30.8	4.0	44.0	20.8	2024	-	L	
V0004	12.6	0.1	214.0	7.7	149.0	6.5	33.5	3.2	160.4	-	607	-	L	
V0005	10.5	0.1	209.1	7.0	130.7	5.2	27.4	5.0	158.4	-	607	-	L	
V0006	12.6	0.1	210.4	7.0	138.1	6.0	29.8	2.1	130.8	33.2	607	-	L	
V0007	10.5	0.1	0.0	0.0	0.0	0.0	33.8	2.2	175.4	-	607	-	M	
V0008	12.6	0.1	0.0	0.0	0.0	0.0	30.3	1.9	358.1	19.2	607	-	L	
V0009	12.6	0.1	0.0	0.0	0.0	0.0	30.3	1.9	157.7	-	607	-	L	
V0010	10.5	0.2	361.3	15.3	189.1	7.1	31.4	2.4	279.4	-	607	-	L	
V0011	12.6	0.2	363.5	14.2	182.5	6.2	28.1	5.7	279.4	-	607	-	L	
V0012	10.5	0.2	366.1	14.5	199.1	7.4	31.7	2.9	341.8	-	607	-	L	
V0013	12.6	0.01	54.9	1.8	76.2	4.4	32.0	4.1	31.1	2.2	2024	-	L	
V0014	10.5	0.01	53.3	1.6	70.2	3.8	25.4	3.3	29.8	-	2024	-	L	
V0015	12.6	0.01	53.8	1.6	74.0	4.0	31.6	4.0	32.1	1.4	2024	-	L	
V0016	10.5	0.1	207.0	6.6	132.8	5.0	28.9	3.9	219.3	73.5	607	-	L	
V0017	12.6	0.1	208.0	7.2	138.3	5.6	31.3	2.1	180.0	0.5	607	-	L	
V0018	10.5	0.1	211.4	7.2	141.6	6.2	31.9	2.0	211.2	55.6	607	-	L	
V0019	12.6	0.2	362.2	13.9	181.2	6.4	26.2	3.1	288.1	-	607	-	L	
V0020	10.5	0.2	360.2	12.0	179.2	5.6	25.2	3.8	385.7	40.0	607	-	L	
V0021	12.6	0.2	358.8	12.8	186.6	6.7	30.5	2.1	296.6	42.1	607	-	L	
V0022	74.3	0.01	40.3	1.2	61.0	2.7	27.2	3.3	22.3	-	2024	-	L	
V0023	88.9	0.01	0.0	0.0	0.0	0.0	30.8	2.7	22.4	0.3	2024	-	L	
V0024	74.3	0.01	42.7	1.3	73.2	3.3	34.2	2.9	24.1	2.7	2024	-	L	
V0025	88.9	0.1	194.8	5.4	133.6	3.8	31.3	2.8	126.7	0.9	607	-	L	
V0026	74.3	0.1	195.9	5.3	137.5	4.6	33.6	5.3	113.6	6.9	607	-	L	
V0027	88.9	0.1	196.3	4.2	139.4	3.3	30.8	2.5	139.2	18.3	607	-	L	
V0028	74.3	0.2	316.1	9.1	170.5	3.8	32.0	1.9	202.5	24.2	607	-	L	
V0029	88.9	0.2	308.8	4.5	147.6	2.3	27.1	4.0	202.7	6.5	607	-	L	
V0030	74.3	0.2	319.2	6.7	172.1	3.3	33.3	1.8	232.9	33.3	607	-	L	
V0031	190.5	0.01	35.1	0.7	53.2	1.1	23.8	1.9	18.0	2.2	2024	-	L	
V0032	159.1	0.01	37.1	1.0	64.2	1.4	29.1	2.9	25.7	-	2024	-	L	
V0033	190.5	0.01	41.3	0.7	79.2	1.3	34.2	1.7	18.4	-	2024	-	L	
V0034	159.1	0.1	179.4	4.6	126.9	2.2	33.6	2.4	113.5	-	607	-	L	
V0035	190.5	0.1	175.5	1.4	119.2	0.8	32.7	1.8	148.9	25.9	607	-	L	
V0037	159.1	0.2	288.5	6.2	144.5	2.8	29.7	4.0	206.8	9.4	607	-	M	
V0038	190.5	0.2	289.8	4.8	158.9	2.4	32.8	2.2	198.0	-	607	-	M	
V0039	159.1	0.2	289.2	3.3	145.0	2.2	29.3	3.6	198.8	-	607	-	M	
V0040	190.5	0.4	491.3	7.5	225.5	9.3	28.9	4.2	337.1	-	607	-	M	
V0041	159.1	0.4	471.4	16.9	203.5	19.6	33.3	2.9	365.6	-	607	-	M	
V0042	190.5	0.4	490.8	8.5	238.2	8.3	35.6	2.4	368.6	-	607	-	M	
V0043	212.2	0.01	36.0	1.1	67.5	1.4	30.0	4.3	19.2	-	2024	-	L	
V0044	254.1	0.01	42.3	0.8	84.2	1.5	37.5	1.7	17.6	-	4049	-	L	
V0045	212.2	0.01	38.0	0.9	66.4	1.5	30.5	2.0	24.3	-	4049	-	L	
V0046	254.1	0.01	39.5	0.8	72.5	1.2	32.1	3.6	19.6	2.8	4049	-	L	
V0047	212.2	0.1	171.0	2.3	120.9	1.0	33.8	2.8	117.3	10.7	607	-	L	
V0048	254.1	0.1	168.3	2.3	119.0	1.9	32.9	2.5	87.9	3.3	607	-	L	
V0049	212.2	0.1	171.8	2.8	126.7	2.0	36.8	1.9	85.0	-	607	-	L	
V0050	254.1	0.2	269.5	4.1	145.0	2.9	31.4	2.1	146.2	1.8	607	-	L	
V0051	212.2	0.2	288.3	6.1	165.0	4.4	37.5	2.1	201.3	6.8	607	-	L	
V0052	254.1	0.2	271.2	7.1	143.7	4.7	31.3	2.8	166.6	35.4	607	-	L	
V0053	212.2	0.4	481.5	13.6	214.9	5.9	31.7	2.3	298.5	12.1	607	-	M	
V0054	254.1	0.4	449.8	12.2	208.4	7.9	35.3	3.4	234.9	17.2	607	-	M	
V0055	212.2	0.4	483.4	8.0	216.1	4.1	33.4	3.7	351.4	38.0	607	-	M	
V0056	381.3	0.01	38.6	0.7	68.6	1.2	33.0	3.0	20.4	1.9	4049	-	L	
V0057	318.5	0.01	38.0	0.8	69.0	1.2	34.2	2.2	14.0	-	4049	-	L	
V0058	381.3	0.01	36.2	0.7	61.0	1.0	28.4	2.3	18.3	-	4049	-	L	
V0059	318.5	0.1	162.0	3.5	112.1	2.6	32.2	2.2	223.9	101.9	1214	-	L	
V0060	381.3	0.1	157.5	3.3	115.5	2.8	33.0	2.4	99.7	-	1214	-	L	
V0061	318.5	0.1	159.1	3.8	104.9	3.0	28.7	4.6	122.3	10.1	1214	-	L	
V0062	381.3	0.2	262.1	5.7	162.7	11.8	34.1	2.1	145.4	6.5	1214	-	M	
V0063	318.5	0.2	270.3	4.9	158.3	6.0	32.5	2.1	147.7	10.1	1214	-	M	
V0064	381.3	0.2	260.0	4.3	165.6	9.5	35.2	2.7	153.5	-	1214	-	M	
V0065	318.5	0.4	455.6	10.5	272.1	32.2	36.8	2.2	232.3	20.9	1214	-	H	
V0066	381.3	0.4	468.5	9.4	412.3	59.6	34.8	2.1	161.5	-	1214	-	H	
V0067	318.5	0.4	453.9	12.1	253.6	28.6	35.8	4.2	261.3	-	1214	-	H	
V0068	254.1	0.2	299.5	12.7	305.9	103.5	29.8	5.0	212.3	2.5	11136	-	H	
V0301	10.0	0.02	79.0	2.3	85.7	5.3	36.8	3.0	-	-	3324	-	L	
V0302	10.0	0.02	83.5	2.4	97.2	5.9	41.2	2.2	93.1	27.3	3324	-	L	
V0303	10.0	0.02	78.5	2.2	84.9	5.3	34.1	2.8	-	-	3324	-	L	
V0304	10.0	0.06	155.4	4.8	121.7	6.5	40.6	2.4	103.6	6.8	3324	-	L	
V0305	10.0	0.06	154.3	4.9	118.9	6.5	39.9	2.9	-	-	3324	-	L	
V0306	10.0	0.06	156.4	5.0	125.0	6.9	41.3	2.5	-	-	3324	-	L	
V0307	10.0	0.1	$-$0.2	2.3	$-$0.1	1.4	34.4	4.3	-	-	3324	-	L	
V0308	10.0	0.1	223.0	7.2	152.4	7.7	42.2	4.4	-	-	3324	-	L	
V0309	10.0	0.1	223.5	7.8	154.6	8.4	42.4	3.6	164.2	4.2	3324	-	L	
V0310	50.1	0.02	61.7	1.7	71.4	3.3	32.4	4.0	-	-	3324	-	L	
V0311	50.1	0.02	68.2	1.9	95.1	4.7	42.5	1.9	49.3	7.9	3324	-	L	
V0312	50.1	0.02	67.1	1.8	89.8	4.4	40.7	2.4	-	-	3324	-	L	
V0313	50.1	0.06	139.8	4.2	121.3	4.5	38.4	2.9	-	-	3324	-	L	
V0314	50.1	0.06	141.5	4.4	126.7	4.7	40.2	3.6	-	-	3324	-	L	
V0315	50.1	0.06	138.4	4.2	117.8	4.4	38.3	2.7	75.7	3.6	3324	-	L	
V0316	50.1	0.1	211.4	6.2	143.2	3.7	38.7	2.5	-	-	3324	-	L	
V0317	50.1	0.1	210.8	6.2	141.6	3.8	36.7	2.2	-	-	3324	-	L	
V0318	50.1	0.1	210.9	5.9	140.6	3.4	36.3	2.2	152.1	-	3324	-	L	
V0319	10.0	0.1	221.9	7.1	145.8	7.1	37.7	2.4	-	-	3324	-	L	
V0320	19.9	0.01	55.9	3.1	84.5	5.2	40.5	2.8	22.8	-	3324	-	L	
V0321	19.9	0.01	57.2	1.8	85.8	5.1	39.2	2.3	-	-	3324	-	L	
V0322	19.9	0.01	56.2	2.2	84.3	5.0	39.5	2.2	-	-	3324	-	L	
V0323	19.9	0.04	111.7	4.2	108.5	5.9	38.1	3.1	-	-	3324	-	L	
V0324	19.9	0.04	104.3	3.1	87.8	4.4	31.2	3.6	-	-	3324	-	L	
V0325	19.9	0.04	110.5	3.5	104.9	5.6	37.5	2.6	74.5	10.7	3324	-	L	
V0326	19.9	0.1	211.1	6.5	146.8	6.4	37.3	2.0	-	-	3324	-	M	
V0327	19.9	0.1	213.5	6.8	158.1	7.1	42.9	2.2	-	-	3324	-	M	
V0328	19.9	0.1	205.8	6.4	133.0	5.5	33.5	3.5	146.7	-	3324	-	M	
V0329	100.0	0.01	44.1	1.0	81.4	3.3	40.6	2.1	-	-	3324	-	L	
V0330	100.0	0.01	43.5	1.1	77.7	3.1	40.0	2.7	-	-	3324	-	L	
V0331	100.0	0.01	43.6	1.0	78.7	3.1	38.8	2.7	22.8	1.1	3324	-	L	
V0332	100.0	0.04	99.0	2.1	96.0	2.4	33.8	3.3	-	-	3324	-	L	
V0333	100.0	0.04	98.4	2.0	93.9	2.1	34.5	3.2	73.4	13.7	3324	-	L	
V0334	100.0	0.04	102.8	2.4	111.5	3.0	41.0	2.7	-	-	3324	-	L	
V0335	100.0	0.1	203.3	2.9	140.0	2.3	37.9	4.7	-	-	3324	-	M	
V0336	100.0	0.1	200.0	2.3	122.0	1.5	32.5	3.6	180.3	-	3324	-	M	
V0337	100.0	0.1	199.2	2.5	123.6	2.0	32.8	3.2	-	-	3324	-	M	
V0338	39.9	0.01	48.5	1.3	77.5	3.9	42.4	2.2	-	-	3324	-	L	
V0339	39.9	0.01	47.1	1.2	72.6	3.8	39.1	4.8	-	-	3324	-	L	
V0340	39.9	0.01	45.9	1.2	68.2	3.6	35.2	2.4	24.7	2.7	3324	-	L	
V0341	39.9	0.04	102.3	3.0	100.2	4.6	36.7	2.4	-	-	3324	-	L	
V0342	39.9	0.04	103.1	3.0	103.7	4.9	38.0	2.4	-	-	3324	-	L	
V0343	39.9	0.04	100.3	2.9	97.7	4.4	36.0	4.4	87.3	33.7	3324	-	L	
V0344	39.9	0.1	211.9	6.8	152.2	5.1	40.1	3.2	-	-	3324	-	L	
V0345	39.9	0.1	209.0	6.6	143.5	4.4	37.4	2.3	-	-	3324	-	L	
V0346	39.9	0.1	209.3	6.8	143.8	4.9	36.7	2.1	133.4	-	3324	-	L	
V0347	125.0	0.01	35.0	0.7	49.1	1.5	28.6	4.1	-	-	3324	-	L	
V0348	125.0	0.01	42.7	0.9	77.5	2.6	38.3	2.4	19.4	-	3324	-	L	
V0349	125.0	0.01	43.1	0.9	78.8	2.6	38.9	2.3	-	-	3324	-	L	
V0350	125.0	0.04	102.3	2.3	110.9	3.2	37.9	2.4	62.4	-	3324	-	L	
V0351	125.0	0.04	97.7	2.2	93.0	2.5	32.9	2.6	-	-	3324	-	L	
V0352	125.0	0.04	101.5	2.3	107.1	3.0	39.5	4.2	-	-	3324	-	L	
V0353	125.0	0.1	197.3	2.1	141.2	1.7	40.5	3.0	192.0	47.7	3324	-	M	
V0354	125.0	0.1	197.7	2.0	141.2	1.8	40.1	3.1	-	-	3324	-	M	
V0355	125.0	0.1	195.9	2.0	138.9	1.7	39.8	2.4	-	-	3324	-	M	
V0356	19.9	0.02	69.9	1.9	77.8	4.2	36.1	2.8	-	-	3324	-	L	
V0357	19.9	0.02	75.1	2.0	91.4	5.1	40.9	2.1	-	-	3324	-	L	
V0358	19.9	0.02	71.8	1.9	82.0	4.5	35.4	2.0	45.6	13.3	3324	-	L	
V0359	19.9	0.08	170.1	5.1	112.5	4.4	30.7	3.3	-	-	3324	-	L	
V0360	19.9	0.08	174.4	5.4	127.9	6.0	38.8	2.8	124.7	-	3324	-	L	
V0361	19.9	0.08	177.0	5.6	138.3	6.6	42.5	2.4	-	-	3324	-	L	
V0362	19.9	0.15	291.4	9.5	171.8	5.9	38.5	2.7	-	-	3324	-	L	
V0363	19.9	0.15	292.8	9.7	176.4	6.1	39.2	2.3	211.6	-	3324	-	L	
V0364	19.9	0.15	293.2	9.4	172.8	5.5	38.4	3.2	-	-	3324	-	L	
V0365	100.0	0.02	61.0	1.4	80.6	3.0	37.2	2.8	39.4	14.1	3324	-	L	
V0366	100.0	0.02	62.9	1.5	89.9	3.2	37.5	3.0	-	-	3324	-	L	
V0367	100.0	0.02	60.9	1.3	82.5	3.0	35.0	3.1	-	-	3324	-	L	
V0368	100.0	0.08	173.8	3.3	129.3	2.8	39.2	3.4	111.2	5.1	3324	-	M	
V0369	100.0	0.08	175.4	3.1	137.6	2.6	42.5	3.5	-	-	3324	-	M	
V0370	100.0	0.08	174.7	3.2	135.9	2.7	44.6	7.2	-	-	3324	-	M	
V0371	100.0	0.15	265.0	3.0	161.1	2.1	43.3	4.0	-	-	3324	-	M	
V0372	100.0	0.15	265.7	3.0	161.8	2.2	42.6	3.2	-	-	3324	-	M	
V0373	100.0	0.15	264.8	3.0	159.1	2.0	41.1	3.0	315.8	86.7	3324	-	M	
V0374	19.9	0.06	148.2	4.4	122.5	5.6	34.5	2.6	121.5	28.7	3282	-	L	
V0375	19.9	0.06	150.9	4.5	134.9	6.7	40.7	2.1	-	-	3282	-	L	
V0376	19.9	0.06	143.6	4.3	115.4	5.1	32.4	3.1	-	-	3282	-	L	
V0377	19.9	0.12	248.4	7.8	172.7	6.2	40.4	2.2	164.5	10.0	3282	-	M	
V0378	19.9	0.12	240.7	7.3	149.2	4.5	33.0	4.2	-	-	3282	-	M	
V0379	19.9	0.12	245.1	7.8	169.6	6.3	40.7	2.7	-	-	3282	-	M	
V0380	19.9	0.2	366.6	14.0	198.7	9.1	40.9	2.8	237.1	20.2	3282	-	M	
V0381	19.9	0.2	366.3	13.4	198.0	9.8	37.8	2.1	-	-	3282	-	M	
V0382	19.9	0.2	367.6	13.6	190.7	9.5	36.5	3.0	287.4	76.1	3282	-	M	
V0383	70.0	0.06	140.0	3.7	127.8	3.3	41.6	2.2	99.9	17.0	3282	-	L	
V0384	70.0	0.06	$-$7.8	29.5	$-$11.7	27.6	44.5	2.3	-	-	3282	-	L	
V0385	70.0	0.06	139.1	3.2	122.6	2.9	39.4	3.3	-	-	3282	-	L	
V0386	70.0	0.12	237.6	4.0	151.1	4.0	39.4	2.4	330.1	19.4	3282	-	M	
V0387	70.0	0.12	236.2	4.3	149.3	4.4	38.2	2.8	-	-	3282	-	M	
V0388	70.0	0.12	240.0	4.0	162.5	3.9	43.9	2.6	-	-	3282	-	M	
V0389	70.0	0.2	330.7	4.2	167.6	3.3	37.9	3.4	-	-	3282	-	M	
V0390	70.0	0.2	334.4	4.8	181.7	5.8	40.5	2.4	230.3	18.6	3282	-	M	
V0391	70.0	0.2	335.3	4.1	188.1	3.7	41.9	2.0	-	-	3282	-	M	
V0392	19.9	0.06	137.7	3.1	122.0	3.0	39.3	2.4	-	-	3282	-	L	
V0393	50.1	0.04	99.0	2.4	95.8	3.5	34.6	3.5	-	-	3282	-	L	
V0394	50.1	0.04	99.3	2.4	98.8	3.6	36.0	2.8	67.9	-	3282	-	L	
V0395	50.1	0.04	101.3	2.4	107.4	4.0	40.8	3.2	-	-	3282	-	L	
V0396	50.1	0.08	172.2	4.6	129.9	3.5	38.4	3.8	-	-	3282	-	L	
V0397	50.1	0.08	174.5	4.5	141.3	3.8	42.2	2.5	108.7	-	3282	-	L	
V0398	50.1	0.08	166.4	4.6	110.6	3.1	32.4	3.4	-	-	3282	-	L	
V0399	50.1	0.15	281.3	2.7	166.9	2.7	41.7	2.3	-	-	3282	-	M	
V0400	50.1	0.15	279.3	2.9	162.5	3.3	40.8	2.6	177.6	-	3282	-	M	
V0401	50.1	0.15	274.1	2.9	144.9	2.9	34.9	3.3	-	-	3282	-	M	
V0402	70.0	0.04	100.6	2.5	108.0	3.6	40.5	2.5	64.2	2.5	3282	-	L	
V0403	70.0	0.04	99.6	2.4	104.4	3.4	37.7	2.4	-	-	3282	-	L	
V0404	70.0	0.04	98.4	2.2	99.3	3.0	36.2	2.8	-	-	3282	-	L	
V0405	70.0	0.08	168.2	3.5	121.4	2.3	36.6	2.7	110.5	10.9	3282	-	M	
V0406	70.0	0.08	165.7	3.3	113.2	1.8	33.9	3.0	-	-	3282	-	M	
V0407	70.0	0.08	165.7	3.5	115.7	2.2	35.9	2.9	-	-	3282	-	M	
V0408	70.0	0.15	271.8	2.9	149.0	2.2	33.7	4.3	-	-	3282	-	M	
V0409	70.0	0.15	272.6	3.5	158.4	2.8	37.3	2.5	-	-	3282	-	M	
V0410	70.0	0.15	274.1	3.3	160.4	2.6	37.6	2.4	225.8	19.1	3282	-	M	
V0411	70.0	0.1	202.7	4.2	133.0	3.4	36.8	3.0	-	-	3282	-	M	
V0412	70.0	0.1	202.7	4.3	133.7	3.4	38.1	3.1	129.0	4.2	3282	-	M	
V0413	70.0	0.1	203.0	4.3	138.5	3.7	38.6	2.3	-	-	3282	-	M	
V0414	50.1	0.2	337.4	5.8	174.0	6.1	38.5	2.8	445.6	162.9	3282	-	M	
V0415	50.1	0.2	328.4	5.5	143.0	4.3	29.1	4.1	-	-	3282	-	M	
V0416	50.1	0.2	336.8	6.3	174.0	5.5	37.6	2.5	-	-	3282	-	M	
V0417	52.0	0.02	67.8	1.5	89.3	3.7	37.3	2.5	-	-	3435	-	L	
V0418	52.0	0.02	64.6	1.5	79.1	3.2	36.1	2.9	-	-	3435	-	L	
V0419	52.0	0.02	67.3	1.5	89.0	3.6	39.1	2.4	41.0	1.5	3435	-	L	
V0420	52.0	0.08	183.0	4.5	143.0	3.7	39.5	2.3	121.1	32.4	3435	-	L	
V0421	52.0	0.08	180.6	4.5	138.5	3.5	38.1	2.2	-	-	3435	-	L	
V0422	52.0	0.08	180.7	4.3	138.1	3.4	37.8	1.9	-	-	3435	-	L	
V0423	52.0	0.12	247.7	4.7	153.7	2.8	38.9	2.8	184.6	37.6	3435	-	L	
V0424	52.0	0.12	247.9	4.6	152.1	2.7	38.8	4.0	-	-	3435	-	L	
V0425	52.0	0.12	248.4	4.7	162.1	3.2	41.1	2.6	-	-	3435	-	L	
V0426	162.4	0.02	65.9	0.9	98.2	1.7	41.9	2.2	-	-	3435	-	L	
V0427	162.4	0.02	63.1	0.9	86.9	1.5	37.8	3.2	35.2	2.2	3435	-	L	
V0428	162.4	0.02	58.1	0.7	68.4	1.1	30.5	4.3	-	-	3435	-	L	
V0429	162.4	0.08	171.5	1.6	137.6	2.2	39.6	2.4	119.0	15.0	3435	-	M	
V0430	162.4	0.08	169.4	1.3	128.1	1.5	37.7	3.0	-	-	3435	-	M	
V0431	162.4	0.08	171.5	1.6	138.5	2.3	41.0	1.9	-	-	3435	-	M	
V0432	162.4	0.12	222.3	1.9	146.5	2.3	38.1	2.8	-	-	3435	-	M	
V0433	162.4	0.12	222.6	2.4	150.6	3.0	39.4	2.3	151.1	4.7	3435	-	M	
V0434	162.4	0.12	221.7	2.0	150.3	2.6	42.0	2.2	-	-	3435	-	M	
V0435	60.0	0.02	65.4	1.4	86.1	3.4	36.6	2.4	-	-	3435	-	L	
V0436	60.0	0.02	63.8	1.3	81.7	3.1	36.6	3.1	-	-	3435	-	L	
V0437	60.0	0.02	66.3	1.4	88.9	3.5	39.0	2.6	39.5	-	3435	-	L	
V0438	60.0	0.1	211.8	3.6	136.2	2.3	37.3	3.1	-	-	3435	-	M	
V0439	60.0	0.1	212.3	3.7	140.0	2.3	37.2	2.1	-	-	3435	-	M	
V0440	60.0	0.1	214.3	3.7	145.3	2.7	39.8	2.9	117.3	-	3435	-	M	
V0441	60.0	0.2	353.5	5.6	191.6	5.3	40.4	3.2	-	-	3435	-	M	
V0442	60.0	0.2	354.2	5.6	192.5	3.9	39.9	3.0	285.5	71.6	3435	-	M	
V0443	60.0	0.2	353.4	5.5	193.8	3.1	39.4	2.7	-	-	3435	-	M	
V0444	60.0	0.02	65.0	1.3	86.3	3.4	39.9	6.3	-	-	3435	-	L	
V0445	60.0	0.02	67.8	1.4	96.3	3.9	41.3	2.0	37.6	3.8	3435	-	L	
V0446	79.9	0.02	66.5	1.4	95.8	3.5	41.2	2.4	-	-	3435	-	L	
V0447	79.9	0.1	205.6	3.6	126.9	3.7	32.8	3.9	-	-	3435	-	M	
V0448	79.9	0.1	213.0	3.4	148.4	3.6	39.5	3.0	181.6	49.5	3435	-	M	
V0449	79.9	0.1	212.0	3.4	149.3	3.7	41.1	3.7	-	-	3435	-	M	
V0450	79.9	0.2	337.1	4.0	171.6	2.1	37.7	3.4	-	-	3435	-	M	
V0451	79.9	0.2	340.5	4.6	186.4	2.9	41.8	2.8	-	-	3435	-	M	
V0452	79.9	0.2	339.0	4.0	179.4	2.4	38.9	2.1	222.4	9.3	3435	-	M	
V0453	79.9	0.02	64.0	1.4	88.2	3.2	36.6	2.8	-	-	3435	-	L	
V0454	79.9	0.02	66.4	1.4	96.5	3.5	40.8	2.6	39.5	8.6	3435	-	L	
V0455	150.0	0.1	196.3	1.4	137.2	1.3	38.5	2.9	141.2	15.0	3435	-	M	
V0456	150.0	0.1	195.9	1.8	134.3	1.5	36.4	2.3	-	-	3435	-	M	
V0457	150.0	0.1	200.1	1.6	152.6	1.7	42.9	2.9	-	-	3435	-	M	
V0458	199.9	0.1	192.7	1.6	137.2	2.8	38.2	2.8	-	-	3435	-	M	
V0459	199.9	0.1	190.5	1.1	142.5	1.8	40.6	2.7	145.4	19.7	3435	-	M	
V0460	199.9	0.1	188.3	1.5	121.4	2.2	32.7	3.7	-	-	3435	-	M	
V0461	400.1	0.01	41.2	0.2	74.3	0.4	35.5	4.5	18.5	1.2	3435	-	L	
V0462	400.1	0.01	37.8	0.2	61.4	0.3	31.7	2.9	-	-	3435	-	L	
V0463	400.1	0.01	40.6	0.2	72.6	0.5	35.9	4.4	-	-	3435	-	L	
V0464	400.1	0.02	61.1	0.4	84.0	0.7	36.7	3.1	-	-	3435	-	L	
V0465	400.1	0.02	63.8	0.4	94.0	0.8	40.9	2.4	-	-	3435	-	L	
V0466	400.1	0.02	62.7	0.5	90.3	0.6	38.2	2.6	30.6	3.2	3435	-	L	
V0467	400.1	0.03	80.5	0.8	96.6	0.8	37.0	2.7	-	-	3435	-	L	
V0468	400.1	0.03	81.2	0.7	98.4	0.8	38.7	3.2	42.7	0.3	3435	-	L	
V0469	400.1	0.03	82.9	0.6	108.1	0.7	42.5	2.4	-	-	3435	-	L	
V0470	400.1	0.04	98.6	1.0	109.0	1.3	38.2	3.2	-	-	3435	-	M	
V0471	400.1	0.04	99.8	0.6	116.0	0.9	41.9	2.9	52.8	1.0	3435	-	M	
V0472	400.1	0.04	97.2	1.0	101.6	1.7	38.4	3.4	-	-	3435	-	M	
V0473	500.0	0.01	42.2	0.6	76.6	0.6	37.7	3.2	-	-	3435	-	L	
V0474	500.0	0.01	44.3	0.7	83.6	0.7	39.1	3.6	19.2	5.2	3435	-	L	
V0475	500.0	0.01	43.2	0.6	81.2	0.7	37.6	2.4	-	-	3435	-	L	
V0476	500.0	0.02	64.0	0.9	91.6	0.9	38.4	2.6	-	-	3435	-	L	
V0477	500.0	0.02	66.0	0.6	101.1	0.8	42.3	2.5	-	-	3435	-	L	
V0478	500.0	0.02	65.6	0.6	98.7	1.0	39.9	2.3	54.9	31.8	3435	-	L	
V0479	500.0	0.1	234.1	14.0	603.5	23.7	39.9	2.3	86.4	50.6	3435	-	H	
V0480	40.0	0.06	139.5	3.6	115.9	4.1	34.7	2.8	-	-	3348	-	L	
V0481	40.0	0.06	142.7	3.8	127.8	4.8	39.7	2.4	109.5	15.4	3348	-	L	
V0482	40.0	0.06	140.4	3.7	121.2	4.4	36.9	2.5	-	-	3348	-	L	
V0483	40.0	0.15	290.2	7.9	174.8	8.7	42.1	3.1	-	-	3348	-	M	
V0484	40.0	0.15	286.8	7.5	173.3	10.3	40.0	3.4	177.7	-	3348	-	M	
V0485	40.0	0.15	285.3	7.4	162.7	9.8	38.0	2.4	-	-	3348	-	M	
V0486	40.0	0.2	349.7	6.5	178.7	9.7	33.2	4.4	-	-	3348	-	M	
V0487	40.0	0.2	352.3	5.2	169.1	6.0	33.4	3.1	-	-	3348	-	M	
V0488	40.0	0.2	353.1	6.2	180.6	8.8	37.2	3.3	233.7	-	3348	-	M	
V0489	125.0	0.06	141.9	2.2	123.3	2.0	37.7	3.2	-	-	3348	-	M	
V0490	125.0	0.06	141.7	2.3	122.8	2.3	37.9	3.3	88.5	8.6	3348	-	M	
V0491	125.0	0.06	141.9	2.2	125.2	2.1	39.0	3.2	98.9	16.1	3348	-	M	
V0492	125.0	0.15	258.3	2.6	150.5	1.8	37.1	4.0	-	-	3348	-	M	
V0493	125.0	0.15	256.5	2.6	137.3	1.7	34.6	3.2	-	-	3348	-	M	
V0494	125.0	0.15	259.2	2.6	154.2	1.9	39.3	2.7	171.6	2.7	3348	-	M	
V0495	125.0	0.2	322.3	4.9	166.6	5.0	35.4	2.4	-	-	3348	-	M	
V0496	125.0	0.2	324.0	5.3	176.1	5.0	38.6	3.3	296.9	135.6	3348	-	M	
V0497	125.0	0.2	321.7	4.1	173.4	3.6	42.9	4.6	-	-	3348	-	M	
V0498	100.0	0.06	143.9	3.1	136.8	3.7	46.8	2.8	-	-	3348	-	M	
V0499	100.0	0.06	142.5	3.4	132.2	4.0	40.5	3.2	147.5	51.8	3348	-	M	
V0500	100.0	0.06	140.9	3.1	128.7	3.6	39.3	3.0	-	-	3348	-	M	
V0501	100.0	0.12	229.3	2.0	147.5	1.4	39.1	3.0	156.1	4.4	3348	-	M	
V0502	100.0	0.12	227.1	1.9	142.5	1.6	37.7	2.9	-	-	3348	-	M	
V0503	100.0	0.12	229.4	1.9	149.9	1.6	38.1	2.9	-	-	3348	-	M	
V0504	100.0	0.2	325.2	4.1	172.5	2.3	39.4	4.0	-	-	3348	-	M	
V0505	100.0	0.2	329.4	4.1	177.2	2.7	41.0	2.2	241.5	2.0	3348	-	M	
V0506	100.0	0.2	323.9	3.8	164.0	2.4	35.9	2.7	-	-	3348	-	M	
V0507	150.0	0.06	138.2	1.4	118.3	1.1	36.5	3.0	-	-	3348	-	M	
V0508	150.0	0.06	139.8	1.4	130.6	1.5	41.0	2.8	109.3	21.5	3348	-	M	
V0509	150.0	0.06	137.4	1.3	117.1	1.2	37.3	3.0	-	-	3348	-	M	
V0510	150.0	0.12	218.6	1.7	144.3	1.7	39.0	2.4	149.9	13.8	3348	-	M	
V0511	150.0	0.12	219.6	1.8	149.3	1.9	41.2	2.3	-	-	3348	-	M	
V0512	150.0	0.12	216.9	1.7	137.5	1.5	35.4	2.2	-	-	3348	-	M	
V0513	150.0	0.2	324.2	4.7	176.0	7.3	36.0	2.9	-	-	3348	-	M	
V0514	150.0	0.2	325.1	5.8	185.3	7.9	37.8	2.8	276.6	51.4	3348	-	M	
V0515	150.0	0.2	326.2	5.2	184.0	7.3	37.3	2.1	-	-	3348	-	M	
V0516	150.0	0.02	63.6	1.0	91.0	1.9	38.3	2.5	38.5	11.3	3348	-	L	
V0517	150.0	0.02	61.6	0.8	83.2	1.5	34.8	3.6	-	-	3348	-	L	
V0518	150.0	0.02	63.4	0.8	89.0	1.6	36.2	3.9	-	-	3348	-	L	
V0519	150.0	0.03	85.4	1.2	105.7	1.8	40.0	2.8	38.8	0.5	3348	-	L	
V0520	150.0	0.03	87.1	1.2	108.3	1.9	38.1	4.1	-	-	3348	-	L

**Table 18:** Experimental results Ck45 orthogonal cutting tests

Test	$v_c$	*f*	$F_c$	$\sigma _{F_c}$	$F_f$	$\sigma _{F_f}$	$r_n$	$\sigma _{r_n}$	$h_{avg}$	$\sigma _{h_{avg}}$	$l_{cut}$	$T_{chip}$	Wear	Force
	$(m\!/\!min)$	(*mm*/*rev*)	(*N*)	(*N*)	(*N*)	(*N*)	$(\mu m)$	$(\mu m)$	$(\mu m)$	$(\mu m)$	(*mm*)	(*K*)		measurement
Test	$v_c$	*f*	$F_c$	$\sigma _{F_c}$	$F_f$	$\sigma _{F_f}$	$r_n$	$\sigma _{r_n}$	$h_{avg}$	$\sigma _{h_{avg}}$	$l_{cut}$	$T_{chip}$	Wear	Force
	$(m\!/\!min)$	(*mm*/*rev*)	(*N*)	(*N*)	(*N*)	(*N*)	$(\mu m)$	$(\mu m)$	$(\mu m)$	$(\mu m)$	(*mm*)	(*K*)		measurement
V0069	10.1	0.01	52.1	11.0	31.6	6.7	33.9	1.6	-	-	6587	-	L	
V0070	10.1	0.01	47.0	7.1	28.4	4.2	35.5	2.3	71.1	-	6587	360	L	
V0071	10.1	0.01	47.7	9.5	28.4	5.7	39.5	4.9	-	-	6587	-	L	
V0072	10.1	0.1	325.1	26.7	185.5	19.3	42.3	2.4	228.6	-	1317	360	L	
V0073	10.1	0.1	312.1	38.8	175.8	20.0	39.2	3.0	190.2	-	1317	360	L	
V0074	10.1	0.1	310.3	25.2	173.0	15.9	39.0	3.8	231.3	-	1317	360	L	
V0075	10.1	0.2	509.0	9.2	303.2	27.0	42.4	2.0	439.0	-	1317	360	L	
V0076	10.1	0.2	543.9	39.7	306.9	30.7	39.2	4.0	367.9	-	1317	360	M	
V0077	10.1	0.2	543.7	39.9	298.6	32.0	42.4	2.3	516.4	-	1317	360	M	
V0078	10.1	0.2	542.9	32.6	297.3	28.2	33.2	4.7	444.3	-	1317	360	L	
V0079	70.1	0.01	38.5	9.5	24.3	6.4	41.2	2.5	69.0	-	4391	360	L	
V0080	70.1	0.01	38.0	9.0	23.7	5.6	36.3	4.6	-	-	6587	360	L	
V0081	70.1	0.01	37.0	9.8	23.3	6.2	42.4	2.5	90.4	-	21959	360	L	
V0082	70.1	0.1	283.7	14.6	233.6	25.6	40.6	2.6	-	-	1317	250	L	
V0083	70.1	0.1	329.6	8.9	319.8	11.2	39.5	3.6	-	-	1976	250	M	
V0084	70.1	0.1	379.7	4.0	401.0	5.7	38.2	4.1	395.5	-	4391	300	M	
V0085	70.1	0.1	387.3	2.9	412.2	2.9	43.8	2.3	414.8	4.3	4391	290	M	
V0086	70.1	0.2	612.8	9.9	511.3	2.7	38.1	3.5	-	-	2195	290	M	
V0087	70.1	0.2	617.0	8.1	511.5	0.7	41.4	2.5	679.2	-	2195	290	M	
V0088	70.1	0.2	614.7	8.5	511.5	0.7	41.4	2.5	606.5	-	2195	290	M	
V0089	70.1	0.2	614.3	8.3	544.9	14.6	36.0	2.6	-	-	2195	290	M	
V0090	150.0	0.01	34.4	5.3	$-$169.7	4.0	39.8	2.5	57.7	14.2	21959	360	L	
V0091	150.0	0.01	36.5	5.9	25.0	4.3	41.7	2.1	44.0	-	21959	360	L	
V0092	150.0	0.01	36.3	6.4	24.9	4.6	39.5	2.5	37.9	2.2	21959	360	L	
V0093	150.0	0.1	344.4	1.2	334.9	1.6	41.5	3.5	318.0	-	4391	290	M	
V0094	150.0	0.1	346.8	2.3	340.2	3.8	43.4	2.0	346.2	-	4391	290	M	
V0095	150.0	0.1	342.9	2.3	332.4	3.6	36.1	3.0	353.2	-	4391	290	M	
V0096	150.0	0.2	549.0	3.0	410.3	4.1	40.6	2.5	512.8	-	2195	290	M	
V0097	150.0	0.2	544.6	5.0	401.9	9.7	44.7	2.1	529.6	-	2195	360	M	
V0098	150.0	0.2	544.8	3.7	402.0	6.1	41.3	2.2	409.5	36.7	2195	340	M	
V0099	150.0	0.4	876.9	20.6	470.4	39.7	42.9	2.5	724.3	-	1097	320	M	
V0100	150.0	0.4	920.4	5.9	515.7	4.5	40.0	2.5	801.9	96.8	2195	340	M	
V0101	150.0	0.4	899.5	15.1	498.8	23.7	42.0	2.9	638.6	-	4940	340	M	
V0102	30.0	0.02	72.6	27.8	45.2	16.3	40.5	2.9	-	-	3440	360	L	
V0103	30.0	0.02	71.6	24.5	43.9	14.2	39.2	2.9	-	-	3440	360	L	
V0104	30.0	0.02	70.2	24.1	43.2	14.3	40.2	2.7	244.0	-	3440	360	L	
V0105	30.0	0.06	188.3	20.8	108.6	14.2	42.4	2.4	-	-	3440	360	L	
V0106	30.0	0.06	170.6	29.0	96.9	16.4	36.0	3.9	108.3	-	3440	360	L	
V0107	30.0	0.06	166.2	32.6	95.5	18.1	42.3	2.1	-	-	3440	360	L	
V0108	30.0	0.15	377.0	29.3	216.1	19.9	38.3	2.9	242.4	-	3440	360	L	
V0109	30.0	0.15	370.1	27.3	209.4	20.3	42.9	2.3	257.9	-	3440	360	L	
V0110	30.0	0.15	377.1	26.8	218.9	18.8	39.9	2.7	221.5	22.9	3440	360	M	
V0111	100.0	0.02	64.0	18.6	41.2	12.1	40.5	2.4	-	-	3440	360	L	
V0112	100.0	0.02	64.1	19.9	41.9	12.9	38.2	4.9	104.6	-	3440	360	L	
V0113	100.0	0.02	63.2	12.6	41.2	8.3	42.4	3.0	48.4	-	3440	360	L	
V0114	100.0	0.06	198.4	2.9	177.9	4.6	41.6	2.3	-	-	3440	320	M	
V0115	100.0	0.06	196.9	2.3	175.5	3.9	38.1	4.5	-	-	3440	290	M	
V0116	100.0	0.06	195.8	2.7	172.6	4.8	37.3	3.7	161.5	-	3440	320	M	
V0117	100.0	0.15	455.2	6.2	405.4	12.7	38.4	3.5	506.9	21.2	3440	300	M	
V0118	100.0	0.15	461.8	6.5	420.1	11.5	37.2	4.0	-	-	3440	360	M	
V0119	100.0	0.15	476.3	8.1	443.7	14.2	39.5	3.0	-	-	3440	360	M	
V0120	30.0	0.04	126.3	25.2	72.6	15.5	38.2	2.6	-	-	3440	360	L	
V0121	30.0	0.04	127.7	23.3	75.3	13.3	40.5	3.1	117.7	14.8	3440	360	L	
V0122	30.0	0.04	121.7	35.2	71.5	20.1	35.2	4.5	-	-	3440	360	L	
V0123	30.0	0.1	262.5	23.4	152.9	15.5	43.1	2.1	-	-	3440	360	L	
V0124	30.0	0.1	253.2	26.7	144.5	16.8	39.0	2.9	-	-	3440	360	L	
V0125	30.0	0.1	258.1	25.7	147.2	15.7	38.4	2.5	147.3	-	3440	360	L	
V0126	30.0	0.2	488.6	26.6	289.1	23.2	39.6	3.2	-	-	3440	240	M	
V0127	30.0	0.2	501.3	28.5	307.1	30.0	44.0	2.1	448.1	78.9	3440	240	M	
V0128	30.0	0.2	493.3	25.7	296.3	25.5	40.6	3.5	-	-	3440	240	M	
V0129	100.0	0.04	122.9	8.3	91.6	9.0	43.4	2.1	109.1	15.7	3440	250	L	
V0130	100.0	0.04	120.1	10.7	85.4	8.7	31.6	3.3	-	-	3440	360	L	
V0131	100.0	0.04	113.6	12.7	79.9	11.4	36.5	4.7	-	-	3440	240	L	
V0132	100.0	0.1	350.9	3.1	360.2	5.4	39.2	3.5	-	-	3440	320	M	
V0133	100.0	0.1	350.0	2.4	355.7	5.5	37.6	2.3	379.1	-	3440	320	M	
V0134	100.0	0.1	353.6	1.8	364.8	2.8	38.3	2.7	-	-	3440	320	M	
V0135	100.0	0.2	565.3	10.5	459.0	19.1	38.5	6.2	-	-	3440	340	M	
V0136	100.0	0.2	552.9	12.3	444.3	24.6	44.3	5.2	-	-	3440	320	M	
V0137	100.0	0.2	565.9	9.2	461.8	16.3	40.4	3.2	572.9	-	3440	340	M	
V0138	30.0	1.0	1590.6	61.8	1417.5	170.1	0.0	0.0	-	-	3440	-	H	
V0139	50.1	0.02	67.9	25.9	41.2	15.0	38.9	3.7	145.6	-	3308	360	L	
V0140	50.1	0.02	73.1	22.6	45.3	13.3	39.9	3.3	59.4	-	3308	360	L	
V0141	50.1	0.02	68.0	28.1	42.1	17.0	40.4	2.6	-	-	3308	360	L	
V0142	50.1	0.06	164.3	40.3	99.1	23.0	38.4	3.3	-	-	3308	360	L	
V0143	50.1	0.06	167.2	29.7	100.9	16.6	35.2	5.2	191.2	-	3308	360	L	
V0144	50.1	0.06	158.0	40.8	94.6	23.1	36.6	2.6	-	-	3308	360	L	
V0145	50.1	0.15	506.7	10.7	487.4	16.5	42.8	2.6	-	-	3308	240	M	
V0146	50.1	0.15	511.1	6.1	493.2	9.5	43.3	2.5	614.2	-	3308	280	M	
V0147	50.1	0.15	504.7	7.7	483.2	15.1	38.1	2.8	-	-	3308	300	M	
V0148	150.0	0.02	73.5	4.9	59.7	4.3	36.3	2.6	-	-	3308	260	L	
V0149	150.0	0.02	68.2	7.9	51.6	6.2	39.3	2.9	-	-	3308	360	L	
V0150	150.0	0.02	69.2	6.3	53.7	5.5	41.0	4.3	64.3	2.0	3308	240	L	
V0151	150.0	0.06	224.4	1.8	239.6	3.0	37.2	2.1	211.7	-	3308	340	M	
V0152	150.0	0.06	226.2	1.4	244.7	2.0	38.2	3.3	-	-	3308	340	M	
V0153	150.0	0.06	224.5	1.4	239.9	3.0	40.8	1.9	-	-	3308	340	M	
V0154	150.0	0.15	428.6	2.9	353.1	4.1	35.3	2.9	303.8	-	3308	340	M	
V0155	150.0	0.15	425.4	2.3	345.2	3.0	35.4	7.9	-	-	3308	340	M	
V0156	150.0	0.15	430.4	3.6	356.7	4.7	37.9	2.4	-	-	3308	300	M	
V0157	50.1	0.04	113.1	28.8	65.8	16.2	38.2	2.6	-	-	3308	360	L	
V0158	50.1	0.04	122.7	23.1	73.0	13.4	37.8	4.4	114.1	22.1	3308	360	L	
V0159	50.1	0.04	114.3	40.4	68.6	23.4	43.5	4.8	-	-	3308	360	L	
V0160	50.1	0.1	262.1	19.9	168.6	18.4	42.2	3.0	-	-	3308	240	M	
V0161	50.1	0.1	261.7	20.0	167.3	17.0	39.5	2.0	-	-	3308	240	M	
V0162	50.1	0.1	257.4	18.4	160.1	14.1	33.6	3.0	253.0	-	3308	240	M	
V0163	50.1	0.2	654.4	6.4	623.0	10.6	42.4	2.4	-	-	3308	290	M	
V0164	50.1	0.2	651.7	5.2	615.7	8.6	43.2	2.2	720.5	-	3308	340	M	
V0165	50.1	0.2	649.6	5.3	613.7	8.6	42.1	2.9	520.6	-	3308	340	M	
V0166	150.0	0.04	141.8	5.2	131.9	11.9	33.6	3.1	-	-	3308	300	M	
V0167	150.0	0.04	137.9	3.7	128.9	10.2	40.4	2.1	121.3	-	3308	340	M	
V0168	150.0	0.04	139.7	5.7	133.5	14.6	40.5	1.8	170.1	-	3308	320	M	
V0169	150.0	0.1	325.3	3.9	312.0	6.1	35.4	2.9	234.0	-	3308	340	M	
V0170	150.0	0.1	322.4	6.1	307.5	10.7	36.3	2.7	-	-	3308	300	M	
V0171	150.0	0.1	326.6	6.6	313.6	11.5	40.5	2.7	351.0	-	3308	340	M	
V0172	150.0	0.2	517.5	7.9	373.6	13.5	32.7	3.6	-	-	3308	340	M	
V0173	150.0	0.2	519.2	4.3	377.3	5.5	40.3	4.5	406.8	-	3308	340	M	
V0174	150.0	0.2	520.0	12.2	380.3	20.9	40.7	2.8	410.9	-	3308	320	M	
V0175	150.0	0.02	77.3	3.7	77.2	1.7	36.0	3.0	80.3	5.6	165404	-	M	
V0176	10.0	0.02	80.9	15.4	48.6	8.9	39.0	2.4	46.8	-	3256	360	L	
V0177	10.0	0.02	81.9	16.3	49.3	9.5	40.6	2.8	74.4	-	3256	360	L	
V0178	10.0	0.02	80.8	16.4	48.3	9.2	37.2	2.7	-	-	3256	360	L	
V0179	10.0	0.06	195.0	29.5	111.5	15.5	43.5	1.8	241.6	-	3256	360	L	
V0180	10.0	0.06	190.9	18.7	106.3	10.8	33.9	2.7	-	-	3256	360	L	
V0181	10.0	0.06	189.1	28.3	104.9	15.4	35.1	3.4	-	-	3256	360	L	
V0182	10.0	0.15	404.7	24.7	221.7	18.4	41.3	2.2	-	-	3256	360	L	
V0183	10.0	0.15	409.8	22.6	225.3	18.9	35.8	2.4	-	-	3256	360	L	
V0184	10.0	0.15	414.3	23.3	233.3	19.9	40.3	2.0	272.5	21.5	3256	360	L	
V0185	69.9	0.02	62.9	7.8	36.8	5.1	30.0	3.5	-	-	3256	360	L	
V0186	69.9	0.02	66.1	22.0	40.6	13.8	36.4	2.5	68.7	12.3	3256	360	L	
V0187	69.9	0.02	66.7	12.3	40.9	7.6	40.9	2.3	-	-	3256	360	L	
V0188	69.9	0.06	167.1	17.3	109.0	12.7	37.5	4.8	-	-	3256	360	M	
V0189	69.9	0.06	167.0	16.7	109.4	11.7	39.9	2.8	150.3	22.4	3256	360	M	
V0190	69.9	0.06	166.2	15.1	107.8	10.7	36.1	3.5	-	-	3256	360	M	
V0191	69.9	0.15	501.5	5.3	491.3	7.8	39.3	2.8	463.7	-	3256	300	M	
V0192	69.9	0.15	497.3	3.7	485.0	6.1	32.9	4.6	-	-	3256	300	M	
V0193	69.9	0.15	501.2	3.6	490.2	6.3	39.5	3.2	-	-	3256	300	M	
V0194	199.9	0.01	36.9	3.3	26.0	2.5	33.5	3.2	27.2	4.7	3256	360	L	
V0195	199.9	0.01	39.5	3.8	28.3	2.9	40.5	3.8	-	-	3256	360	L	
V0196	199.9	0.01	39.5	3.2	28.4	2.8	39.5	3.1	-	-	3256	360	L	
V0197	199.9	0.04	159.1	1.0	171.3	1.6	40.8	3.1	-	-	3256	290	M	
V0198	199.9	0.04	158.0	1.2	170.2	1.8	33.0	4.1	166.2	-	3256	290	M	
V0199	199.9	0.04	159.3	0.9	172.3	1.1	38.3	3.7	-	-	3256	290	M	
V0200	199.9	0.08	269.7	2.8	261.2	4.1	41.6	4.0	260.9	4.4	3256	360	M	
V0201	199.9	0.08	268.2	2.5	257.7	3.5	36.3	3.6	287.7	-	3256	360	M	
V0202	199.9	0.08	273.3	2.6	267.9	4.2	40.9	2.8	-	-	3256	360	M	
V0203	300.0	0.01	49.6	0.9	51.7	1.2	35.8	3.4	-	-	3256	290	M	
V0204	300.0	0.01	51.5	1.2	55.9	1.8	38.6	2.7	47.7	3.3	3256	360	M	
V0205	300.0	0.01	51.2	1.2	55.6	1.7	37.8	3.0	52.7	3.2	3256	360	M	
V0206	300.0	0.04	146.9	3.3	150.4	3.4	43.5	2.0	-	-	3256	360	M	
V0207	300.0	0.04	149.2	3.2	152.9	5.3	41.7	2.5	134.6	-	3256	360	M	
V0208	300.0	0.04	149.9	2.4	152.9	3.5	36.6	2.9	-	-	3256	360	M	
V0209	300.0	0.08	260.1	2.7	233.5	1.9	42.4	3.0	-	-	3256	360	M	
V0210	300.0	0.08	259.7	1.4	233.0	1.0	43.3	2.5	246.9	-	3256	360	M	
V0211	300.0	0.08	257.5	1.8	227.2	2.1	40.4	2.3	-	-	3256	360	M	
V0212	10.0	0.01	45.2	14.2	28.6	8.3	36.9	2.9	-	-	3256	360	L	
V0213	10.0	0.01	46.0	7.4	28.7	4.6	40.5	2.6	31.2	12.2	3256	360	L	
V0214	10.0	0.01	44.9	7.8	27.6	4.6	35.4	2.8	-	-	3256	360	L	
V0215	10.0	0.04	144.6	27.7	84.1	16.0	39.1	2.9	86.8	16.5	3256	360	L	
V0216	10.0	0.04	145.4	27.6	85.1	15.2	41.6	3.4	-	-	3256	360	L	
V0217	10.0	0.04	138.0	30.8	79.1	16.8	39.6	2.8	-	-	3256	360	L	
V0218	10.0	0.08	249.5	20.2	142.7	12.1	36.3	3.4	-	-	3256	360	L	
V0219	10.0	0.08	249.5	17.4	142.6	11.3	36.7	2.5	157.6	64.0	3256	360	L	
V0220	10.0	0.08	244.7	18.2	138.7	12.0	36.7	2.5	179.5	-	3256	360	L	
V0221	349.9	0.01	49.7	0.5	49.6	0.4	33.5	3.9	-	-	3256	360	L	
V0222	349.9	0.01	56.3	0.5	61.1	0.4	42.6	2.0	-	-	3256	360	L	
V0223	349.9	0.01	54.0	0.5	56.0	0.4	39.8	1.9	36.5	3.8	3256	360	L	
V0224	349.9	0.04	152.1	1.1	156.1	1.6	36.2	2.5	-	-	3256	360	M	
V0225	349.9	0.04	152.1	0.8	154.1	1.5	34.9	2.2	143.2	-	3256	360	M	
V0226	349.9	0.04	153.4	2.0	158.2	2.7	38.7	3.1	-	-	3256	360	M	
V0227	349.9	0.08	250.3	2.3	213.0	2.1	40.6	2.6	-	-	3256	360	M	
V0228	349.9	0.08	245.8	2.0	203.6	2.3	36.5	2.2	216.3	-	3256	360	M	
V0229	349.9	0.08	242.0	3.0	198.0	2.8	34.6	3.2	-	-	3256	360	M	
V0230	250.1	0.02	83.6	0.5	82.3	0.4	43.0	1.8	-	-	3256	320	M	
V0231	250.1	0.02	81.6	0.5	79.3	0.5	39.8	2.5	64.0	5.4	3256	300	M	
V0232	250.1	0.02	82.5	0.7	80.6	0.5	41.6	3.0	-	-	3256	300	M	
V0233	250.1	0.06	207.7	2.4	202.2	3.9	39.9	3.5	-	-	3256	360	M	
V0234	250.1	0.06	213.0	2.8	212.8	4.1	41.3	2.4	192.1	7.5	3256	360	M	
V0235	250.1	0.06	206.2	2.3	198.2	3.8	33.3	3.7	-	-	3256	360	M	
V0236	250.1	0.1	304.0	1.8	256.9	2.8	38.6	2.2	312.8	-	3256	360	M	
V0237	250.1	0.1	304.5	2.8	258.5	4.0	39.5	2.3	-	-	3256	360	M	
V0238	250.1	0.1	304.6	2.6	258.2	3.2	39.2	2.8	-	-	3256	360	M	
V0239	349.9	0.02	87.8	0.7	93.5	0.9	36.2	2.5	91.4	19.6	3256	360	M	
V0240	349.9	0.02	88.7	0.9	94.4	1.0	33.6	2.9	-	-	3256	360	M	
V0241	349.9	0.02	89.3	1.3	97.2	1.6	35.2	3.0	-	-	3256	360	M	
V0242	349.9	0.06	207.1	2.1	194.6	2.5	38.2	3.4	-	-	3256	360	M	
V0243	349.9	0.06	208.6	2.4	199.6	3.0	39.7	2.4	-	-	3256	360	M	
V0244	349.9	0.06	204.3	2.2	187.9	3.0	34.7	3.0	215.3	-	3256	360	M	
V0245	349.9	0.1	285.0	2.6	216.7	1.8	40.1	2.5	263.8	-	3256	360	M	
V0246	349.9	0.1	283.0	3.5	212.8	5.4	37.7	2.6	-	-	3256	360	M	
V0247	349.9	0.1	283.3	3.1	215.1	3.8	39.5	2.7	-	-	3256	360	M	
V0248	349.9	0.15	$-$0.0	0.1	$-$0.0	0.1	33.6	2.8	-	-	3256	360	M	
V0249	349.9	0.15	369.7	4.8	229.6	6.0	35.2	3.1	346.4	-	3256	360	M	
V0250	349.9	0.15	375.2	3.3	236.9	4.2	38.4	3.0	-	-	3256	360	M	
V0251	499.9	0.02	$-$0.0	0.1	$-$0.0	0.1	40.3	2.3	58.0	0.4	3256	360	M	
V0252	50.0	0.01	39.7	13.2	26.8	7.9	34.3	3.8	-	-	3260	360	L	
V0253	50.0	0.01	38.0	9.0	24.2	5.6	40.0	2.4	69.3	13.2	3260	360	L	
V0254	50.0	0.01	37.8	7.4	24.4	4.7	40.4	3.3	-	-	3260	360	L	
V0255	50.0	0.08	211.2	24.2	128.1	15.2	36.0	2.8	168.3	21.5	3260	360	M	
V0256	50.0	0.08	210.5	26.3	128.9	16.8	38.3	2.1	152.9	-	3260	360	L	
V0257	50.0	0.08	209.5	29.4	128.9	18.0	38.1	2.3	-	-	3260	360	M	
V0258	50.0	0.12	320.4	19.8	234.3	33.8	40.2	2.3	-	-	3260	200	M	
V0259	50.0	0.12	325.1	16.7	241.1	29.7	41.2	2.5	339.0	-	3260	200	M	
V0260	50.0	0.12	322.7	20.0	238.8	36.0	40.5	2.0	-	-	3260	200	M	
V0261	250.0	0.01	51.1	0.5	53.9	0.7	35.6	3.1	35.2	-	3260	300	L	
V0262	250.0	0.01	52.6	1.1	57.3	1.3	41.6	2.1	-	-	3260	300	L	
V0263	250.0	0.01	49.3	0.9	51.4	1.3	35.5	2.9	-	-	3260	300	L	
V0264	250.0	0.08	250.4	1.8	223.6	2.6	32.0	3.1	-	-	3260	360	M	
V0265	250.0	0.08	255.7	2.7	234.4	3.4	37.0	2.1	-	-	3260	360	M	
V0266	250.0	0.08	259.8	2.9	241.9	3.9	40.6	2.4	249.9	-	3260	360	M	
V0267	250.0	0.12	335.9	2.9	265.3	3.3	41.7	2.2	-	-	3260	360	M	
V0268	250.0	0.12	330.6	1.5	253.5	1.8	33.3	3.2	-	-	3260	360	M	
V0269	250.0	0.12	334.6	1.8	262.0	2.2	38.3	2.8	336.2	-	3260	360	M	
V0270	50.0	0.18	0.0	0.0	0.0	0.0	38.0	2.3	-	-	2173	360	M	
V0271	50.0	0.18	566.4	8.5	521.5	9.9	36.6	2.6	404.6	29.5	2173	360	M	
V0272	50.0	0.18	569.1	8.6	527.6	12.9	38.7	2.7	-	-	2173	360	M	
V0273	50.0	0.18	572.7	8.9	536.3	13.1	36.0	2.9	-	-	2173	360	M	
V0274	50.0	0.22	678.0	7.3	614.7	12.0	38.5	2.4	-	-	2173	360	M	
V0275	50.0	0.22	678.0	9.8	615.2	12.3	37.6	2.5	683.3	-	2173	360	M	
V0276	50.0	0.22	679.5	13.3	612.1	24.7	40.1	1.9	-	-	2173	360	M	
V0277	200.0	0.02	84.4	1.8	83.5	2.3	38.2	2.3	-	-	3260	300	M	
V0278	200.0	0.02	81.1	1.1	80.3	1.1	40.1	2.6	70.0	6.6	3260	300	M	
V0279	200.0	0.02	79.8	1.0	77.9	1.3	36.2	3.6	-	-	3260	300	M	
V0280	200.0	0.06	213.6	1.9	220.6	3.3	38.8	2.6	198.3	6.5	3260	360	M	
V0281	200.0	0.06	213.1	1.4	218.9	2.4	38.9	2.3	-	-	3260	360	M	
V0282	200.0	0.06	215.6	1.4	224.1	2.6	38.8	3.0	-	-	3260	360	M	
V0283	200.0	0.1	307.6	2.5	275.4	4.3	35.9	2.8	-	-	3260	360	M	
V0284	200.0	0.1	308.3	2.3	272.1	3.6	37.1	3.6	-	-	3260	360	M	
V0285	200.0	0.1	310.3	3.0	279.7	4.3	37.9	2.3	325.5	88.9	3260	360	M	
V0286	450.0	0.02	86.5	1.2	91.1	0.6	40.0	2.7	65.4	7.7	3260	360	M	
V0287	450.0	0.02	86.5	1.2	92.9	0.9	41.1	2.3	-	-	3260	360	M	
V0288	450.0	0.02	86.2	1.5	92.9	1.6	40.6	2.3	-	-	3260	360	M	
V0289	450.0	0.06	193.1	2.6	167.8	1.9	38.0	2.4	142.9	2.5	3260	360	M	
V0290	450.0	0.06	196.0	3.6	173.7	2.1	40.3	2.2	-	-	3260	360	M	
V0291	450.0	0.06	190.1	2.1	162.1	1.8	34.4	3.6	-	-	3260	360	M	
V0292	450.0	0.1	265.1	3.2	183.9	2.9	41.8	2.1	-	-	3260	300	M	
V0293	450.0	0.1	263.6	2.2	180.2	1.2	37.5	2.8	237.7	-	3260	300	M	
V0294	450.0	0.1	261.6	3.6	178.5	3.8	37.4	2.1	-	-	3260	300	M	
V0295	50.0	0.2	644.5	4.6	611.4	6.9	34.3	3.5	-	-	3260	360	M	
V0296	50.0	0.2	643.6	4.6	608.4	6.1	37.7	2.7	503.9	-	3260	360	M	
V0297	50.0	0.2	642.6	14.5	596.9	23.9	38.3	2.5	-	-	3260	360	M	
V0298	500.0	0.1	259.6	1.9	169.5	2.6	37.2	3.8	-	-	3260	280	M	
V0299	500.0	0.1	259.4	2.5	173.2	3.6	40.2	3.4	240.7	-	3260	280	M	
V0300	500.0	0.1	260.3	1.5	174.6	2.3	42.0	2.4	228.2	8.4	3260	280	M	

## Appendix 4. Kienzle parameters

The Kienzle coefficients are given as a function of cutting speed for Ti6Al4V in Table 19 and for Ck45 in 20.

**Table 19:** Ti6Al4V: Kienzle coefficients for feed and cutting force estimations

$v_c$	(*m*/*min*)	11	13	20	40	50	52	60	70
$k_{c1.1}$	$(N/mm^2)$	1299	1264	1265	1253	1061	1156	1146	1156
$m_c$	$(-)$	0.21	0.22	0.22	0.22	0.28	0.27	0.27	0.24
$k_{f1.1}$	$(N/mm^2)$	413	333	421	352	274	328	300	282
$m_f$	$(-)$	0.51	0.63	0.54	0.61	0.7	0.66	0.69	0.69
$v_c$	(*m*/*min*)	74	80	89	100	125	150	159	162
$k_{c1.1}$	$(N/mm^2)$	941	1084	891	932	962	998	869	1027
$m_c$	$(-)$	0.32	0.28	0.34	0.33	0.31	0.3	0.31	0.28
$k_{f1.1}$	$(N/mm^2)$	282	267	177	226	283	283	235	303
$m_f$	$(-)$	0.69	0.73	0.89	0.77	0.69	0.7	0.72	0.67
$v_c$	(*m*/*min*)	191	212	254	318	381	400	500	
$k_{c1.1}$	$(N/mm^2)$	881	859	749	712	663	803	662	
$m_c$	$(-)$	0.32	0.32	0.37	0.36	0.38	0.35	0.41	
$k_{f1.1}$	$(N/mm^2)$	255	248	199	186	205	329	280	
$m_f$	$(-)$	0.71	0.72	0.8	0.78	0.75	0.66	0.73

**Table 20:** Ck45: Kienzle coefficients for feed and cutting force estimations

$v_c$	(*m*/*min*)	10	30	50	70	100	150	200
$k_{c1.1}$	$(N/mm^2)$	1935	1803	1417	1729	1672	1707	1716
$m_c$	$(-)$	0.19	0.17	0.51	0.35	0.32	0.28	0.26
$k_{f1.1}$	$(N/mm^2)$	996	995	654	1156	809	765	1030
$m_f$	$(-)$	0.23	0.2	0.96	0.55	0.65	0.6	0.45
$v_c$	(*m*/*min*)	250	300	350	450			
$k_{c1.1}$	$(N/mm^2)$	1794	1851	1575	1368			
$m_c$	$(-)$	0.22	0.22	0.27	0.29			
$k_{f1.1}$	$(N/mm^2)$	1268	1446	1024	587			
$m_f$	$(-)$	0.31	0.29	0.38	0.53			
